# Gtr/Ego-independent TORC1 activation is achieved through a glutamine-sensitive interaction with Pib2 on the vacuolar membrane

**DOI:** 10.1371/journal.pgen.1007334

**Published:** 2018-04-26

**Authors:** Hirofumi Ukai, Yasuhiro Araki, Shintaro Kira, Yu Oikawa, Alexander I. May, Takeshi Noda

**Affiliations:** 1 Graduate School of Frontier Biosciences, Osaka University, Osaka, Japan; 2 Graduate School of Dentistry, Osaka University, Osaka, Japan; 3 Research Center of Cell Biology, Institute of Innovative Research, Tokyo Institute of Technology, Yokohama, Japan; University of Wisconsin Madison, UNITED STATES

## Abstract

TORC1 is a central regulator of cell growth in response to amino acids. The role of the evolutionarily conserved Gtr/Rag pathway in the regulation of TORC1 is well-established. Recent genetic studies suggest that an additional regulatory pathway, depending on the activity of Pib2, plays a role in TORC1 activation independently of the Gtr/Rag pathway. However, the interplay between the Pib2 pathway and the Gtr/Rag pathway remains unclear. In this study, we show that Pib2 and Gtr/Ego form distinct complexes with TORC1 in a mutually exclusive manner, implying dedicated functional relationships between TORC1 and Pib2 or Gtr/Rag in response to specific amino acids. Furthermore, simultaneous depletion of Pib2 and the Gtr/Ego system abolishes TORC1 activity and completely compromises the vacuolar localization of TORC1. Thus, the amino acid-dependent activation of TORC1 is achieved through the Pib2 and Gtr/Ego pathways alone. Finally, we show that glutamine induces a dose-dependent increase in Pib2-TORC1 complex formation, and that glutamine binds directly to the Pib2 complex. These data provide strong preliminary evidence for Pib2 functioning as a putative glutamine sensor in the regulation of TORC1.

## Author summary

TORC1 is a central regulator of cell growth in response to amino acids. The evolutionarily conserved Gtr/Rag pathway is a well-established TORC1 regulatory pathway. In this study, we show that two molecular machineries, Pib2 and Gtr/Ego, form distinct complexes with TORC1 in a mutually exclusive manner, implying an exclusive functional relationship between TORC1 and Pib2 or Gtr/Rag in response to various amino acids. We also show that the amino acid-dependent activation of TORC1 is achieved through the Pib2 and Gtr/Ego pathways by anchoring them to the vacuolar membrane. Finally, we show that glutamine binds directly to the Pib2 complex and that glutamine enhances Pib2-TORC1 complex formation. Collectively we provide evidence supporting a role for Pib2 as an element of a putative glutamine sensor.

## Introduction

Cell growth is primarily governed by environmental nutritional conditions [1]. TORC1, a protein complex that is universally conserved among eukaryotes, plays a pivotal role in the cell’s coordinated response to amino acids [2,3]. In the budding yeast, *Saccharomyces cerevisiae*, TORC1 consists of a central protein kinase, Tor1 or Tor2, along with Kog1, Lst8 and Tco89 [2]. When amino acids are available, TORC1 activates anabolic processes such as protein synthesis and suppresses catabolic processes such as autophagy, and these effects are reversed under amino acid or nitrogen starvation conditions [4,5]. The progress of each anabolic or catabolic process is controlled by the phosphorylation status of corresponding TORC1 substrates, such as Sch9 and Atg13 [6,7].

A fundamental but poorly addressed question in the study of TORC1 concerns the mechanism by which amino acid availability is interpreted and results in the activation or deactivation of TORC1. In yeast, the most well-established regulator of TORC1 is the heterodimeric small GTPase complex Gtr1–Gtr2, the orthologue of which is RagA/B–RagC/D in mammals [8,9]. Both Gtr1 and Gtr2 bind to a guanine nucleotide, GTP or GDP, with the GTP/GDP-bound state of each subunit distinct at any given moment [10]. Gtr1–Gtr2 is anchored to the vacuolar membrane via a scaffold known as the Ego protein complex, consisting of Ego1, Ego2, and Ego3 [11–13]. When Gtr1 binds to GTP, it binds to and activates TORC1 [10]. In mammals, the GTP form of RagA/B recruits mTORC1 to the lysosomal membrane, where it encounters the small GTPase, Rheb, which results in the activation of mTORC1 [8]. In contrast, the existence of a similar TORC1 activator corresponding to Rheb remains ambiguous in budding yeast. Recently, multiple amino acid sensor proteins were identified in mammals, namely Sestrin and CASTOR, which are leucine and arginine sensor proteins, respectively [14,15]. However, it remains unclear whether similar amino acid sensors exist in yeast. Recent work has demonstrated that Gtr2 is regulated by its own GTPase-activating protein (GAP), Lst4–Lst7, which is an orthologue of mammalian FNIP–Folliculin and appears to be controlled by amino acid availability [16–18], suggesting that a similar system may exist for Gtr1.

Despite these advances in our understanding of Gtr/Rag-dependent TORC1 regulation, one intriguing observation in particular remains to be accounted for: although TORC1 is essential for cell growth, Δ*gtr1* or Δ*gtr2* mutants show only a very slight defect in growth. Recently, Stracka *et al*. provided an important insight into this apparent paradox, finding that there are two types of TORC1-activating responses determined by the availability of certain amino acids [19]. One of these is the Gtr-dependent response, in which cells exhibit a transient increase in TORC1 activity in response to poor nitrogen sources, exemplified by leucine. Cells also show a rapid and sustained activation of TORC1 that is independent of Gtr in response to preferred nitrogen sources such as glutamine. Thus, two independent mechanisms are thought to activate TORC1 in response to amino acids. Although the specific proteins responsible for the glutamine-responsive system remain unknown, genetic experiments suggest that it could involve the vacuolar membrane protein Pib2. Pib2 was originally identified as a protein with phosphatidylinositol (PtdIns) 3P-binding activity mediated by its own FYVE domain [20]. Kim et al. showed that Pib2 is also involved in TORC1 regulation: the Δ*pib2* mutant exhibits synthetic lethality with Δ*gtr1* and lysosomal membrane permeabilization in response to endoplasmic reticulum membrane stress [21]. Two more recent studies suggested that Pib2 might transduce glutamine signals to TORC1 in parallel to the Gtr/Ego system [22,23]. However, these studies were unable to address several important questions surrounding such a role for Pib2, including whether the amino acid-dependent activation of TORC1 is achieved through the Pib2 and Gtr/Ego pathways alone (i.e., the effect of the simultaneous absence of Pib2 and the Gtr/Ego system on the activity and localization of TORC1); the nature of the molecular mechanism by which Pib2 modulates TORC1 activity; the identity of what senses glutamine; and how glutamine regulates TORC1 activity.

In this study, we provide further characterization of the role of Pib2 in the glutamine-responsive pathway for TORC1 activation independently of the Gtr/Ego system. Our detailed analyses provide three important findings that clarify the function of Pib2. First, we find that Pib2 and Gtr/Ego form distinct complexes with TORC1 in a mutually exclusive manner. Second, our data indicate that simultaneous depletion of Pib2 and the Gtr/Ego system abolishes TORC1 activity and completely compromises the vacuolar localization of TORC1. Thus, the data strongly suggest that amino acid-dependent activation of TORC1 is achieved through the Pib2 and Gtr/Ego pathways alone. Finally, we show that glutamine induces a dose-dependent increase in TORC1 complex formation and that glutamine binds directly to the Pib2 complex. These data suggest that Pib2 plays a role as an integral component of a putative glutamine sensor.

## Results

### Pib2 is a core component of the glutamine-responsive pathway of TORC1 activation

Three recent studies have suggested that Pib2 might transduce glutamine signals to TORC1 in parallel to the Gtr/Ego system [21–23]. We first attempted to validate these findings by adopting an alternative, detailed approach. Genome-wide synthetic genetic array (SGA) analysis showed that the mutation of *PIB2* results in a synthetic growth defect with the TORC1 subunits *TOR1* and *TCO89*, and *SLM4/EGO3*, a component of the Ego complex [24]. Consistent with these previous findings, we also observed synthetic lethality of Δ*pib2* with Δ*gtr1* and Δ*ego1* (S1A Fig). We next investigated the response of Δ*pib2* and Δ*gtr1* cells to supplementation of individual amino acids using an amino-acid prototrophic strain maintained on YMM, a synthetic, minimal medium that allows for the examination of the effect of individual amino acids [19,25]. Cells were grown in YMM medium containing ammonium sulfate as the sole nitrogen source (i.e., in the absence of any amino acids), before being subjected to nitrogen starvation in nitrogen-free YMM for 30 minutes. Under these conditions, the phosphorylation of Sch9 is reduced to a basal level. Next, various amino acids were added, and the phosphorylation of Sch9 was monitored at each time point. In wild-type cells, addition of glutamine immediately activated TORC1 within 1 minute and sustained activation for 30 minutes (S1B Fig) [19]. In Δ*gtr1* cells, activation was delayed by 5 min, and was marginally weaker, but marked phosphorylation was sustained for at least 30 minutes (S1B Fig). The degree of TORC1 activation was strikingly lower in Δ*pib2* cells than in wild-type cells, although minimal activation was observed at 1 minute (S1B Fig). The low level of phosphorylation continued for 15 minutes before a small increase was observed, although this increase was attenuated in comparison to wild-type cells. We also evaluated the effect of glutamine supplementation on the phosphorylation state of Atg13, another TORC1 substrate that is involved in autophagy. Upon amino acid treatment, Atg13 was phosphorylated with slower kinetics than Sch9 in wild type cells (S1C Fig). As we observed for Sch9 phosphorylation, TORC1 stimulation by glutamine was compromised severely in Δ*pib2* and partially in Δ*gtr1* strains (S1C Fig). These results indicate that Pib2 is crucial for the response to glutamine. This conclusion is also complemented by biochemical analyses of TORC1 activity presented in a recent study [22]. On the other hand, our findings with regard to the phosphorylation response to leucine were in marked contrast to the response to glutamine (S1D Fig). First, wild-type cells exhibited a relatively gradual response to the addition of leucine, starting at 1 minute before reaching a peak at 5–7 minutes. Phosphorylation of Sch9 started to decrease at 9 minutes, and was almost completely absent after 30 minutes (S1D Fig). This confirms that leucine-dependent activation of TORC1 is transient, as described previously [19]. Interestingly, this leucine-dependent transient TORC1 activation was abolished in both Δ*gtr1* cells and Δ*pib2* cells (S1D Fig). However, Δ*gtr1* or Δ*pib2* cells grew in YMM medium supplemented only with leucine as the nitrogen source, albeit to a lesser extent than wild type cells (S1E Fig). This finding may be explained by the metabolic derivation of other amino acids from leucine supporting cell growth under these conditions. Thus, both the Pib2- and Gtr/Ego-dependent pathways are critical in the early phase of leucine-dependent TORC1 activation.

### The amino acid-dependent activation of TORC1 is achieved through the Pib2 and Gtr/Ego pathways alone

**Fig 1:**
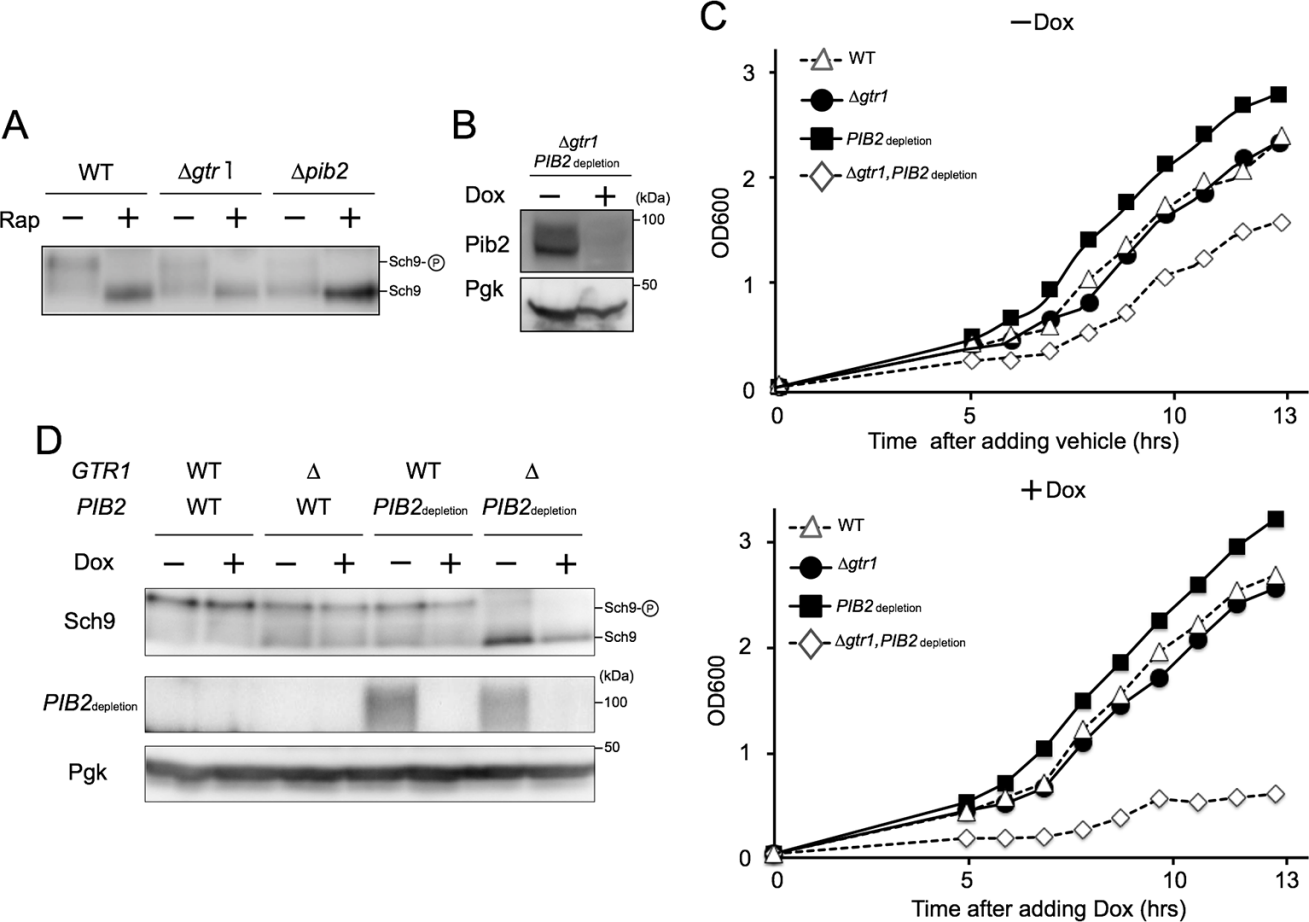
Simultaneous depletion of Pib2 and Gtr1 abolishes TORC1 activity. (A) Cells expressing Sch9-6HA (WT: SKY384, Δ*gtr1*: HUY33, or Δ*pib2*: HUY34) were grown in YPD medium with or without 0.2 μg/ml rapamycin. Lysates were treated with NTCB and subjected to western blotting using an anti-HA antibody. (B) Δ*gtr1* cells harboring *PIB2*_depletion_ (*tet07*-*Ubi-Leu-3HA-PIB2*; HUY50) were grown in SCD supplemented with uracil and treated with ethanol (-Dox) or 4 μg/ml doxycycline (+Dox) for 90 min. Lysates were analyzed by immunoblotting with anti-HA antibody. (C) Cells of the indicated genotypes (WT: BY4741, Δ*gtr1*: YKOL6522, *PIB2*_depletion_: HUY48, Δ*gtr1 PIB2*_depletion_: HUY50) were grown in SCD medium containing ethanol (-Dox) or 4 μg/ml doxycycline (+Dox), and optical density was measured at the indicated time points. (D) Cells of the indicated genotypes (WT: SKY116, Δ*gtr1*: SKY118, *PIB2*_depletion_: HUY51, Δ*gtr1 PIB2*_depletion_: HUY53) expressing Sch9-6HA were grown in SCD containing ethanol (-Dox) or 4 μg/ml doxycycline (+Dox) for 90 min. Lysates were treated with NTCB and analyzed by immunoblotting with the indicated antibodies.

Δ*gtr1* cells exhibit only a limited decrease in TORC1 activity, as determined by the phosphorylation status of the target Sch9 following chemical cleavage with NTCB (Fig 1A) [6]. Likewise, Δ*pib2* cells exhibited only slight decrease in TORC1 activity (Fig 1A). To examine TORC1 activity in the simultaneous absence of Gtr1 and Pib2 proteins, we employed a system that combines the tetracycline-repressible promoter and an N-end rule-based degron that destabilizes the target protein, resulting in complete depletion of the protein [26]. In Δ*gtr1 tetO7-Ubi-Leu-3HA-PIB2 (PIB2*_depletion_*)* cells, the repressible Pib2 mutant protein was mostly depleted from cells 90 minutes after addition of doxycycline to shut off *PIB2* expression (Fig 1B). Even after shutoff, *PIB2*_depletion_ cells grew similarly to wild-type cells, like Δ*gtr1* cells (Fig 1C bottom). However, Δ*gtr1 PIB2*_depletion_ double-mutant cells exhibited minimal proliferation following doxycycline treatment, indicating that Gtr1 and Pib2 play redundant yet essential roles in cell growth (Fig 1C bottom). Remarkably, TORC1 activity was completely abolished in Δ*gtr1 PIB2*_depletion_ double-mutant cells after shutoff, in contrast to the small decrease in TORC1 activity in Δ*gtr1* and *PIB2*_depletion_ single-mutant cells (Fig 1D). Even before doxycycline addition, the level of Pib2 was reduced due to destabilization by the degron, leading to a defect in TORC1 activity and slower growth in combination with Δ*gtr1* (Fig 1C and 1D). Collectively, these data indicate that simultaneous depletion of Pib2 and Gtr1 leads to complete abolition of TORC1 activity, providing the first direct evidence that the amino acid-dependent activation of TORC1 is achieved through the Pib2 and Gtr/Ego pathways alone.

### Pib2 constitutes a complex with TORC1 distinct from the Gtr1-containing complex

**Fig 2:**
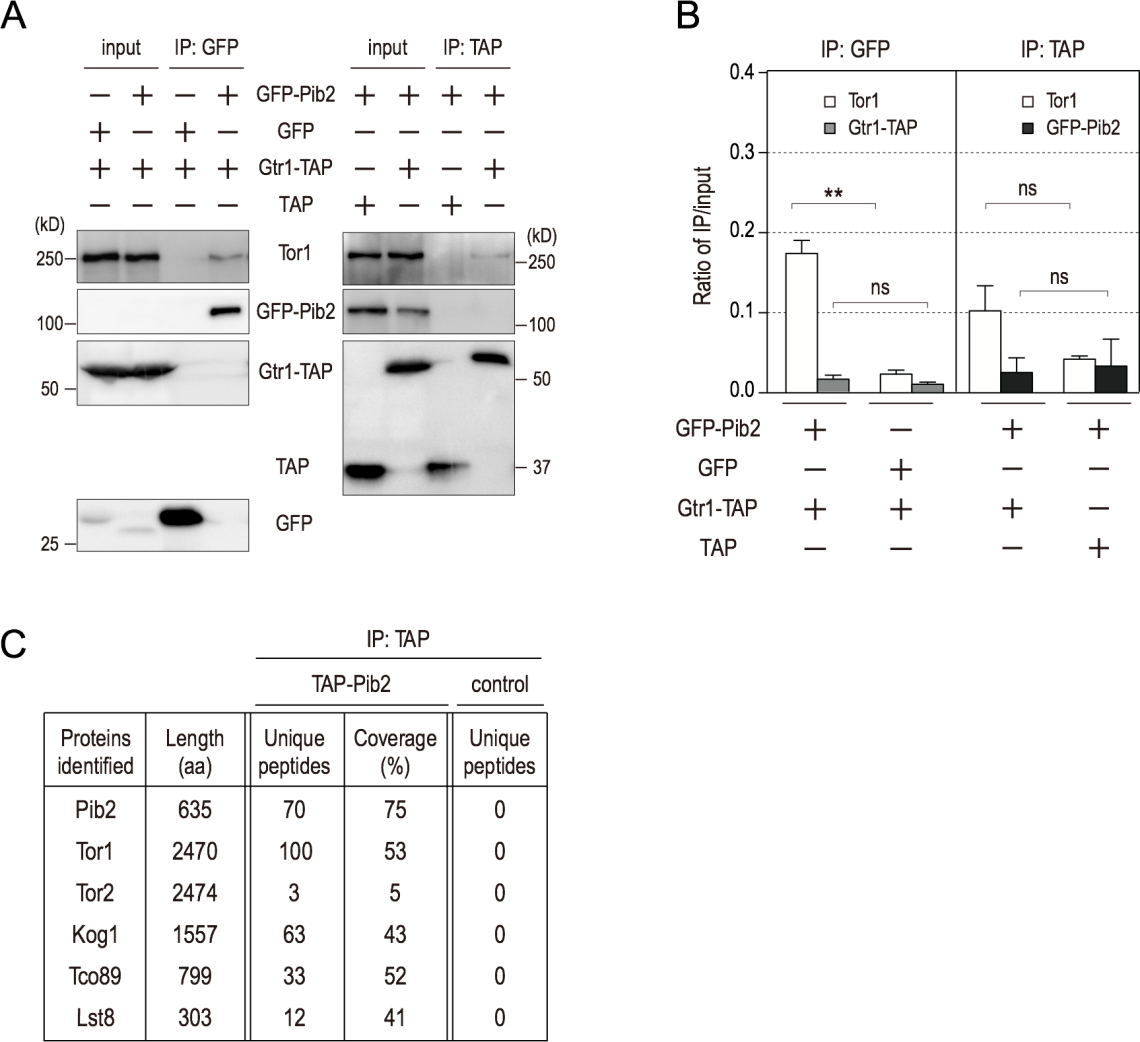
Pib2 interacts with TORC1 independently of Gtr1. (A) Cells expressing the indicated tagged proteins were grown in YPD (*GTR1-TAP GFP-PIB2*: HUY57, *GTR1-TAP GFP*: HUY77, *TAP GFP-PIB2*: HUY58). Extracts were prepared and immunoprecipitated by GFP-Trap or IgG-Dynabeads. Whole-cell extract and precipitated proteins were analyzed by western blotting. (B) Quantification of the ratio of IP/input in Fig 2A. Mean ± SD (n = 3). **p < 0.01, Student’s *t*-test. (C) Proteins detected in TAP-Pib2 (YAY2583) and control (YAY1963) immunoprecipitations by LC-MS/MS analysis are listed.

Next, we sought to determine whether Pib2 is physically associated with TORC1. To this end, we constructed a strain expressing genomically-integrated GFP-Pib2 and Gtr1-TAP under the control of their endogenous promoters in order to ensure expression at physiologically relevant levels. These tags do not have any effect on Pib2 and Gtr1 function, as determined by rapamycin sensitivity (S2A Fig) [13]. Pull-down experiments using magnetic beads conjugated to GFP-binding protein revealed that endogenous Tor1 protein was co-precipitated with GFP-Pib2, indicating that Pib2 is physically associated with TORC1 (Fig 2A left panel). We next investigated Pib2’s relationship with the Gtr complex. However, Gtr1-TAP was not detected in the GFP-Pib2-precipitated fraction (Fig 2A left panel). Conversely, when Gtr1-TAP was pulled down, GFP-Pib2 could not be detected in the precipitated fraction, whereas Gtr1-TAP co-precipitates with Tor1, albeit to a lesser extent than GFP-Pib2 (Fig 2A right panel and 2B. During the process of optimizing conditions to enhance the interaction between Gtr1-TAP and Tor1, we noted that provision of an additional copy of *GTR1-TAP* distinctly increases the protein amount of Tor1 co-precipitated with Gtr1-TAP (S2B Fig right panel, and S2C Fig). Even in this situation, GFP-Pib2 was not detected in the Gtr1-TAP-precipitated fraction (S2B Fig right panel, S2C Fig). In parallel, GFP-Pib2 was co-precipitated with Tor1, but not with Gtr1-TAP (Fig 2B left panel and 2C). We also immunopurified TAP-tagged Pib2 from yeast cells, and co-precipitating proteins were processed for identification using liquid chromatography tandem mass spectrometry (LC–MS/MS). Notably, we found that with the exclusion of common contaminants of proteomic analysis [27], TAP-Pib2 co-immunoprecipitated with all the known components of TORC1, namely Tor1, Tor2, Kog1, Lst8 and Tco89, but not any specific component of the TORC2, Gtr1–Gtr2 or Ego complexes (Fig 2C, S2 Table). These results indicate that Pib2 and Gtr1 independently associate with distinct TORC1 complexes.

### The localization of Pib2 on the vacuole is crucial for TORC1 activation

**Fig 3:**
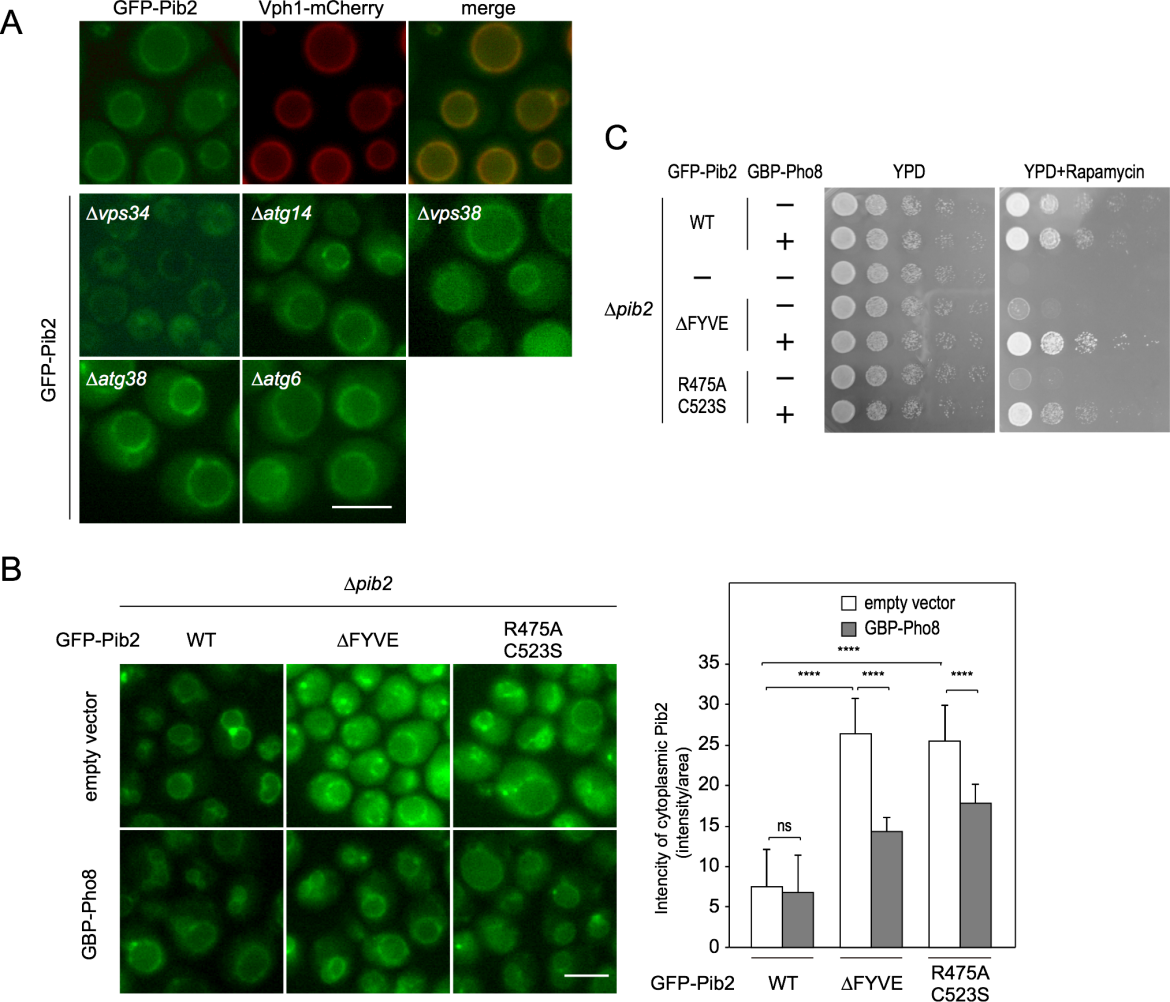
The vacuolar localization of Pib2 mediated by PtdIns3P is required for TORC1 activation. (A) Cells of the indicated genotypes (WT: HUY39, Δ*vps34*: HUY46, Δ*atg14*: HUY65, Δ*vps38*: HUY64, Δ*atg38*: SKY597, Δ*atg6*: SKY598) were grown in SCD supplemented with uracil and adenine, and then shifted to nitrogen-free YMM. Thirty minutes after the shift, glutamine was added to a concentration of 3 mM. After another 30 min, cells were analyzed by fluorescence microscopy. (B) Artificial tethering of GFP-Pib2 with the indicated mutations in the FYVE domain on the vacuolar membrane using the GFP binding protein (GBP) fused to Pho8. Cells (WT: HUY74, ΔFYVE: HUY75, R475AC523S: HUY76) with either pRS314 (empty vector) or pRS314-*GBP-Pho8* (GBP-Pho8) were grown in SCD supplemented with uracil for GFP-Pib2 intensity measurement. Bar, 5 μm. Quantification of the cytoplasmic GFP-Pib2 levels from 30 cells (right panel). Cytoplasmic GFP-Pib2 levels were determined by measuring the intensity and area of GFP fluorescence in the cytoplasm using ImageJ software. Mean ± SE (n = 3). ****p < 0.0001, Student’s *t*-test. (C) Δ*pib2* cells expressing different FYVE mutants of GFP-Pib2 (WT: HUY74, ΔFYVE: HUY75, R475AC523S: HUY76) were serially 10-fold diluted and spotted on YPD plates with or without 0.2 μg/ml rapamycin, and then grown at 30°C for 3 days.

As TORC1 is known to localize to the vacuolar membrane, we next sought to determine the site at which Pib2 fulfills its function. Microscopy of chromosomally tagged GFP-Pib2 expressed from its own promoter revealed that the GFP signal closely co-localizes with Vph1-mCherry, a marker of the vacuolar membrane (Fig 3A). This vacuolar signal was completely abolished in the Δ*vps34* mutant (Fig 3A), in which the sole PtdIns3-kinase, Vps34, is absent, as reported previously [28]. Two Vps34-containing complexes have been experimentally identified in yeast: class I, which consists of Vps34, Vps15, Atg6/Vps30, Atg14, and an Atg38 dimer, functions specifically in autophagy, whereas class II, which consists of Vps34, Vps15, Atg6/Vps30, and Vps38, functions in vacuolar protein sorting [29,30]. However, significant signal persisted on the vacuolar membrane in the absence of the class I complex in Δ*atg14* or Δ*atg38* mutant cells, as well as in the absence of the class II complex in Δ*vps38* mutant cells, and in the absence of both classes in the Δ*atg6/vps30* mutant, indicating that PtdIns3P is generated by a third Vps34 complex distinct from class I or II (Fig 3A). These results are consistent with a recent study that employed an *in vitro* approach to assess TORC1 kinase assay, which found that Atg6 is dispensable for Pib2-dependent TORC1 activation [22].

Pib2 contains a FYVE domain, which is known to bind PtdIns3P [21]. As the localization of Pib2 on the vacuole is totally dependent on PtdIns3P (Fig 3A), we investigated whether the FYVE domain of Pib2 dictates the vacuolar localization of Pib2. We examined this possibility by assessing the subcellular localization of two Pib2 variants, one completely lacking the FYVE domain (ΔFYVE, lacking residues 452–525), whereas the Pib2-R475A C523S variant was mutated at two residues (R475A and C523S), which results in an inability to bind both zinc ions and PtdIns3P [31,32]. Microscopy of these GFP-Pib2 mutants expressed under the control of the endogenous Pib2 promoter revealed that a considerable portion of both GFP-Pib2 mutants remain on the vacuole, although the remainder was observed to disperse throughout the cytoplasm (Fig 3B). This indicates that the vacuolar localization of Pib2 is only partially dependent on its FYVE domain, and that another mechanism may exist for its vacuolar localization. These findings are in contrast with a previous study that used a heterogeneous MET promoter to over-express Pib2 proteins, finding that vacuole localization was entirely dependent upon the FYVE domain [21]. However, cells expressing FYVE mutants were much more sensitive to rapamycin than cells expressing wild-type Pib2, suggesting that TORC1 activity is severely impaired by the appreciable mislocalization of Pib2 (Fig 3C). By constitutively localizing GFP-Pib2 to vacuolar membranes, we were able to further investigate the significance of Pib2 localization in the activation of TORC1. To this end, we devised an experiment employing a fusion protein, GFP-binding protein (GBP)-Pho8 (Vacuolar alkaline phosphatase), which localizes to the vacuolar membrane and exposes GBP to the cytoplasm where it is able to bind GFP [13]. Therefore, if the function of Pib2 requires its vacuolar localization, it should be possible to restore TORC1 activity in cells harboring the GFP-Pib2 FYVE mutants by expressing GBP-Pho8. Enhancer GBP was employed in this experiment, binding to which enhances the fluorescence of bound GFP [33]. However, this means that the recruitment of Pib2 to the vacuole results in an increased fluorescence signal, preventing quantitative determination. We therefore followed the change in cytosolic GFP signal, which decreases as Pib2 localizes to the vacuole, to determine the localization of GFP-Pib2 FYVE mutant proteins. As expected, the stable expression of GBP-Pho8 led to a reduced level of cytoplasmic GFP-Pib2, reflecting an increased fraction of GFP-Pib2 localization to the vacuolar membrane (Fig 3B). Importantly, protein levels of the Pib2 FYVE mutants are comparable–indeed marginally lower–than that of wild type Pib2, and are not influenced by the presence of GBP-Pho8 (S3 Fig). In addition, mobility shift of FYVE mutants was observed only in the presence of GBP-Pho8, hinting at a role for activated TORC1 in Pib2 modification at the vacuolar membrane (S3 Fig, left panel). The constitutive localization of Pib2 FYVE mutants further resulted in the reactivation of TORC1, as indicated by rapamycin sensitivity of GBP-Pho8-expressing strains in comparison to cells lacking GBP-Pho8 (Fig 3C). Thus, we conclude that the vacuolar localization of Pib2 is necessary for the execution of its function in TORC1 activation.

### Alterations in Pib2 localization are linked to TORC1 regulation

**Fig 4:**
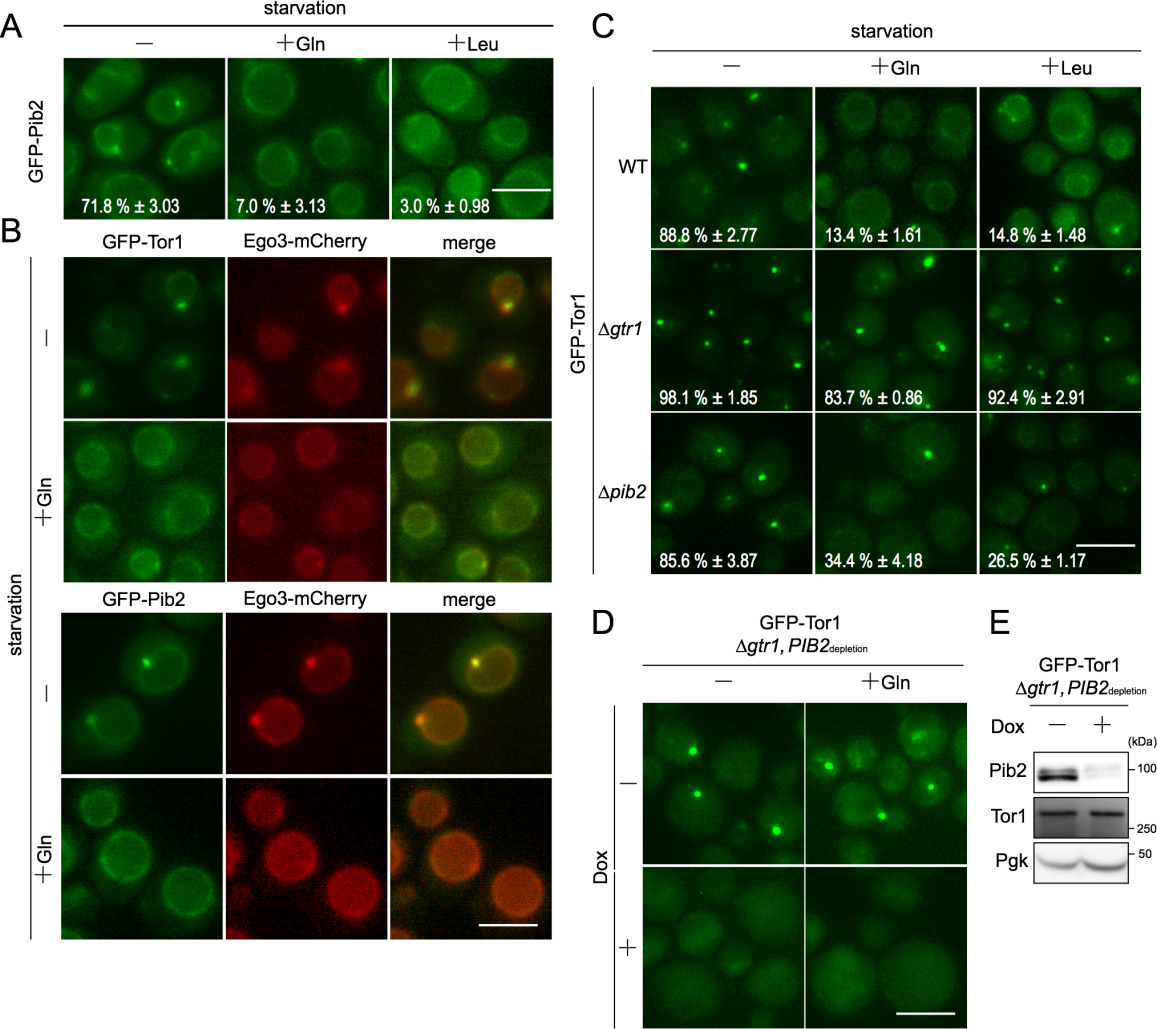
Pib2 and Gtr1 are necessary to localize TORC1 to lysosomal membranes. (A) Cells expressing GFP-Pib2 (HUY45) were cultured and analyzed by fluorescence microscopy as in Fig 3A with the addition of glutamine or leucine. The percentage of cells with vacuolar membrane-associated puncta is shown for each image. Statistical data are shown as Mean ± SE from 100–200 cells of four independent experiments. (B) Cells expressing GFP-Pib2 (HUY41) or GFP-Tor1 (SKY596) and Ego3-mCherry were cultured and analyzed by fluorescence microscopy as in Fig 3A. Bar, 5 μm. (C) Cells expressing GFP-Tor1 in WT (SKY222) and the indicated genotypes (Δ*gtr1*: SKY278, Δ*pib2*: HUY6) were cultured and analyzed by fluorescence microscopy, as in Fig 3A. Statistical data are shown as mean ± SE from 100–200 cells of four independent experiments. (D) Δ*gtr1* cells harboring *PIB2*_depletion_ (*tet07*-*Ubi-Leu-3HA-PIB2*) and *GFP-TOR1* (HUY70) were grown in SCD supplemented with uracil and adenine before the addition of 4 μg/ml doxycycline or ethanol. 30 min after treatment with doxycycline or ethanol, cells were shifted to nitrogen-free YMM with either doxycycline or ethanol. 30 min after the shift, glutamine was added at a concentration of 3 mM. After another 30 min, cells were analyzed by fluorescence microscopy. Bar, 5 μm. (E) Cells harboring *PIB2*_depletion_ and GFP-Tor1 in Δ*gtr1* (HUY70) were grown in SCD supplemented with uracil and treated with 4 μg/ml doxycycline or ethanol for 90 min. Lysates were analyzed by immunoblotting with anti-HA, anti-Tor1, and anti-Pgk1 antibodies.

In addition to the constitutive vacuolar localization of GFP-Pib2, we also observed the natural formation of vacuole-associated GFP-Pib2 puncta under nitrogen starvation. After addition of either glutamine or leucine, these puncta disappeared (Fig 4A, S4A Fig). Therefore, we investigated the relationship between GFP-Pib2 and GFP-Tor1 puncta [34]. Because tagging of both Pib2 and Tor1 with mCherry provided insufficient signal, we used Ego3-mCherry as a reference, a protein that co-localizes with GFP-Tor1, as previously reported (Fig 4B) [13]. GFP-Pib2 exhibited clear co-localization with Ego3-mCherry both on the vacuolar membrane and in puncta (Fig 4B). These observations provide strong evidence that TORC1-containing Pib2 and Gtr/Ego complexes are localized in close proximity to each other under nitrogen starvation conditions.

### Both Pib2 and the Gtr/Ego complex are required for TORC1 tethering to the vacuolar membrane

In wild-type cells subjected to nitrogen starvation, TORC1 shifts between the vacuolar membrane and vacuole-associated puncta. Following the addition of glutamine to cells starved for nitrogen for 30 minutes, GFP-Tor1 puncta disappeared within 5 minutes, with the protein reassuming its localization at the vacuolar membrane (Fig 4C, S4B Fig, S1 Video). When leucine was added in place of glutamine, an essentially identical shift in localization was observed, although puncta disappeared more slowly (Fig 4C, S4B Fig). The translocation of Tor1 is evidently highly regulated by amino acids.

In Δ*gtr1* cells, GFP-Tor1 constitutively formed puncta, even after the addition of glutamine (Fig 4C, S4B Fig), which is consistent with a previous report [13]. Interestingly, GFP-Pib2 was also observed predominantly as puncta in Δ*gtr1* cells even after the addition of either glutamine or leucine, although limited vacuolar signal persisted (S4C and S4D Fig). This was not because the distribution of PtdIns3P became punctate, as determined by monitoring of GFP-FYVE, a general probe for PtdIns3P (S4E Fig). Therefore, these data suggest that the Gtr system causes TORC1 and Pib2 to spread over the vacuolar membrane in response to amino acids. By contrast, Δ*pib2* cells exhibited a change in GFP-Tor1, as in wild-type cells, albeit to a slightly reduced extent (Fig 4C, S4B Fig).

We next investigated the effect of simultaneous depletion of Pib2 and Gtr1 on the localization of Tor1. Using the same Δ*gtr1 PIB2*_depletion_ (*tetO7-Ubi-Leu-3HA-PIB2*) double-mutant strain described above, we observed the formation of GFP-Tor1 puncta associated with the vacuole (Fig 4D). Upon treatment with doxycycline, GFP-Tor1 dissociated from puncta but did not localize to the vacuolar membrane irrespective of the presence of supplemented amino acid, with GFP-Tor1 remaining diffused throughout the cytoplasm (Fig 4D). Western blotting indicated that the disappearance of GFP-Tor1 puncta was not due to changes in protein abundance (Fig 4E). Taken together, we conclude that, at least under the conditions assessed, the TORC1 complex is tethered to the vacuolar membrane exclusively through the Pib2 and Gtr/Ego pathways.

### Glutamine enhances the Pib2-TORC1 interaction

**Fig 5:**
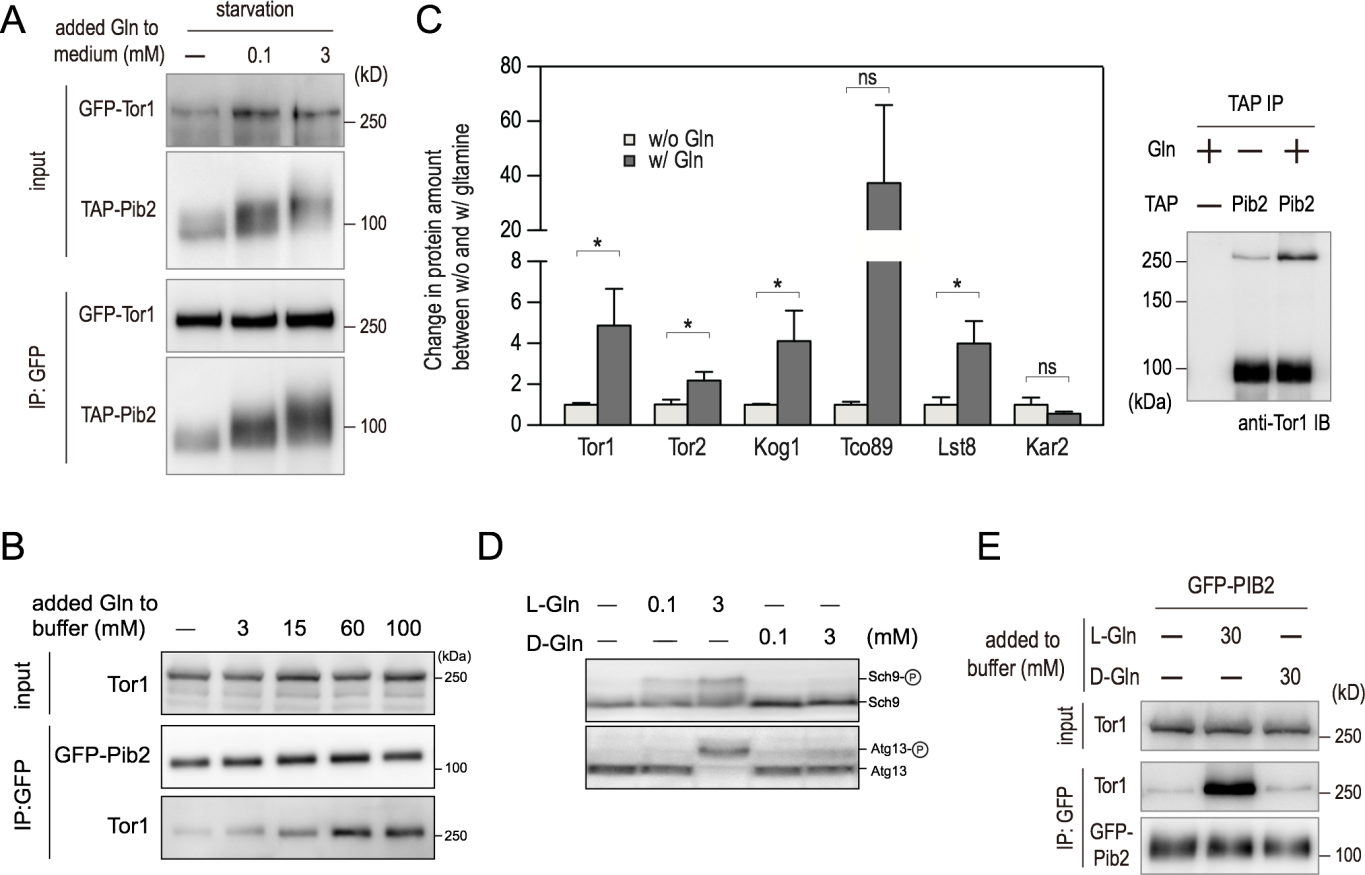
l-glutamine, but not d-glutamine, reinforces the Tor1-Pib2 interaction in cells and *in vitro*. (A) Cells expressing GFP-Tor1 and TAP-Pib2 (HUY71) were grown in YPD and then shifted to nitrogen-free YMM. Glutamine was added at various concentrations 30 min following shift. After another 30 min, extracts were prepared and immunoprecipitated by GFP-Trap. Whole-cell extract (top, “input”) and precipitated proteins (bottom) were analyzed by western blotting. (B) Cells expressing GFP-Pib2 (HUY45) were grown in YPD. Various concentrations of glutamine were added to all buffers used in the experiment, and cell lysates were prepared and analyzed as in (A). (C) Relative abundance of proteins identified in TAP-Pib2 (YAY2583) purifications using buffers with or without 60 mM of glutamine. The relative abundance of each protein is defined as a ratio of emPAI_protein of interest_/emPAI_Pib2_. In order to compare results from multiple experiments, the values for experiments conducted in the absence of glutamine were normalized to 1.0. Statistical data are shown as mean ± SD of three independent experiments. *p < 0.05, Student’s *t*-test. A portion of TAP-Pib2 purifications were subjected to immunoblotting with indicated antibodies (right panel). (D) Cells expressing Sch9-6HA (Wild type: SKY384) were grown in YMM medium supplemented with ammonium sulfate, and then shifted to nitrogen-free YMM. Thirty minutes after the shift, indicated concentrations of either l-glutamine or d-glutamine were added to the cultures. Cells were harvested 10 min (Sch9) or 30 min (Atg13) after glutamine addition. Phosphorylation of Sch9 or Atg13 was monitored by immunoblotting with the indicated antibodies. For the analysis of Sch9 phosphorylation, lysates were treated with NTCB and subjected to western blotting using an anti-HA antibody. (E) Cells expressing GFP-Pib2 (HUY45) were grown in YPD. Different concentrations of either l-glutamine or d-glutamine were added to all buffers used in the experiment, and cell lysates were prepared and analyzed as in (A).

To investigate the molecular mechanism underlying the amino acid dependent activation of TORC1 by Pib2, we examined the effect of nutrient conditions on the association between Pib2 and TORC1. As GFP-Pib2 could not be immunoprecipitated under nitrogen starvation conditions, we instead conducted a pull-down of GFP-Tor1 expressed under the control of its own promoter (Fig 5A). Amino acid deprivation led to a decrease in the amount of Pib2 bound to Tor1 (Fig 5A, S5A Fig). However, this reduction was suppressed by the addition of glutamine in a dose-dependent manner (Fig 5A, S5A Fig). We also observed a mobility shift of GFP-Pib2 in response to glutamine addition (Fig 5A). This shift is dependent on phosphorylation by TORC1 because treatment with either phosphatase or rapamycin led to the collapse of the slower migrating GFP-Pib2 species into a single, faster-migrating protein band (S5B Fig). However, we have not yet assessed the physiological relevance of Pib2 phosphorylation. This immunoprecipitation result prompted us to test the possibility that glutamine might act directly on the interaction between Pib2 and TORC1. Consistent with this possibility, addition of glutamine to all buffers used in the pull-down experiment efficiently improved the co-precipitation of Tor1 by GFP-Pib2 in a dose-dependent manner (Fig 5B, S5C Fig). This effect was specific to glutamine: the presence of leucine did not enhance the interaction (S5D and S5E Fig). Semi-quantitative LC-MS/MS analyses of co-purified materials with TAP-Pib2 using glutamine-supplemented buffers provided further evidence to support this model (Fig 5C). The abundance of each co-purified protein was quantified by the exponentially modified Protein Abundance Index (emPAI) [35]. This approach again showed that glutamine addition strengthened the interaction between Pib2 with all known components of TORC1 *in vitro*, with the exception of Tco89 (Fig 5C). The lack of significance observed for Tco89 was due to variation between replicate measurements. In spite of this inconsistent result for Tco89, a strong trend of increased co-precipitation in the presence of glutamine is evident. The increase apparently represents specific binding because the amount of Kar2, a common contaminant of proteomic analyses, co-precipitating with Pib2 was not affected by the supplementation of glutamine (Fig 5C).

Although the two stereoisomers of glutamine, l- and d-glutamine, have the same physical and chemical properties, only l-glutamine is synthesized and incorporated into proteins in cells. Re-addition of d-glutamine to amino acid starved cells failed to sustain TORC1 activity as assessed by Sch9 and Atg13 phosphorylation (Fig 5D). Crucially, in clear contrast to l-glutamine, d-glutamine did not effect a dose-dependent increase in Pib2-TORC1 interaction when added to immunoprecipitation buffers, providing further evidence for the physiological significance of Pib2-mediated TORC1 activation (Fig 5E, S5F Fig).

### The interaction of Pib2 with TORC1 is required for its activation by glutamine

**Fig 6:**
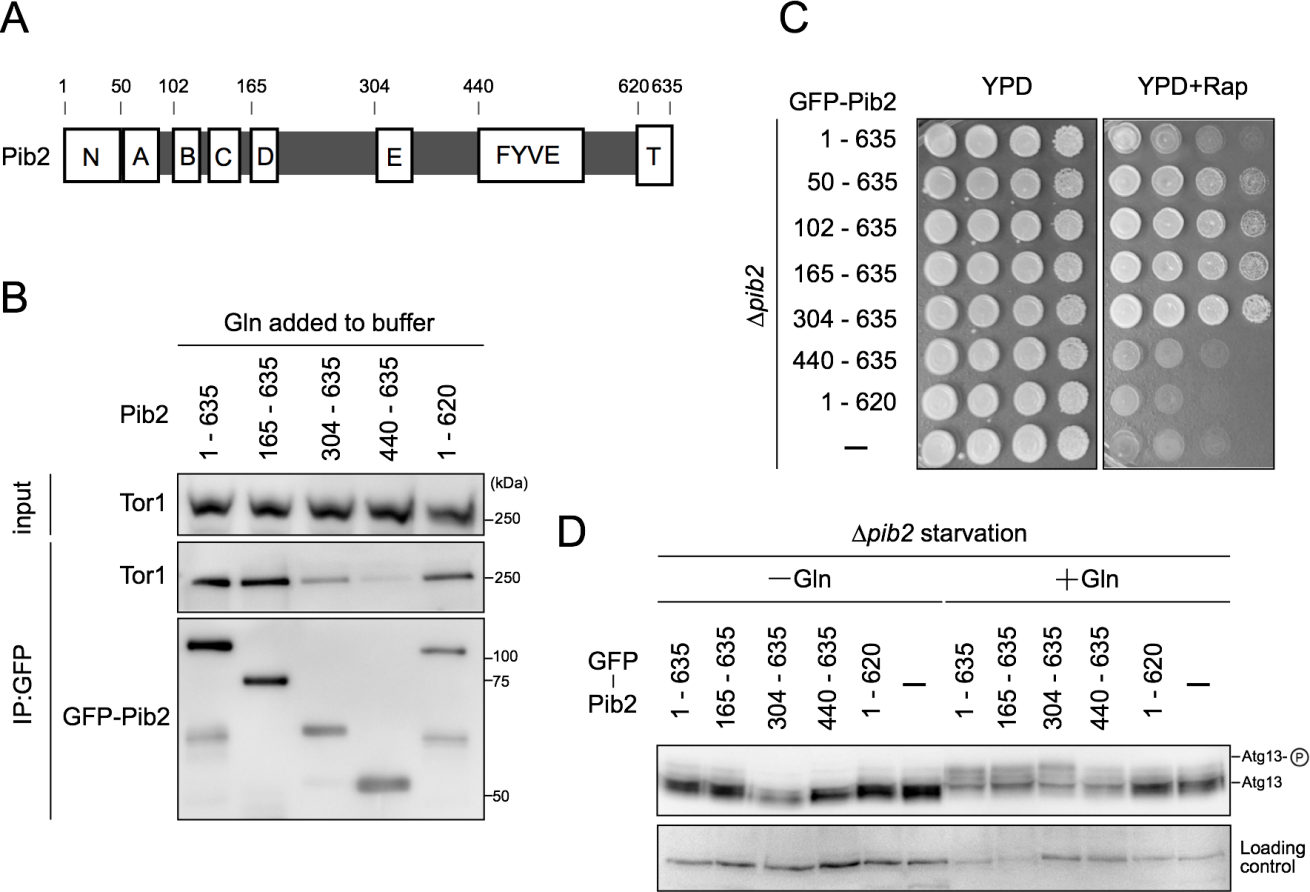
Pib2 interacts with TORC1 for its activation by glutamine. (A) A schematic diagram of Pib2 protein with the defined motifs. (B) Δ*pib2* cells expressing different truncation mutants of GFP-Pib2 (YAY2569, YAY2571, YAY2572, YAY2573, YAY2574) were grown in YPD. Glutamine was added (60 mM) to all buffers used in the experiment, and cell lysates were prepared and immunoprecipitation performed using GFP-Trap. Whole-cell extract (top, “input”) and precipitated proteins (bottom) were analyzed by western blotting. (C) Δ*pib2* cells expressing different truncation mutants of GFP-Pib2 (YAY2569, YAY2575, YAY2570, YAY2571, YAY2572, YAY2573, YAY2574 and HUY29) were serially diluted 10-fold and spotted onto YPD plates with or without 0.2 μg/ml rapamycin, and then grown at 30°C for 3 days. (D) Δ*pib2* cells expressing different truncation mutants of GFP-Pib2 (YAY2569, YAY2571, YAY2572, YAY2573, YAY2574 and HUY29) were grown in SCD supplemented with uracil, and then shifted to nitrogen-free YMM. Thirty minutes after the shift, glutamine (3 mM) was added. After another 30 min, cells were harvested. Phosphorylation of Atg13 was examined by immunoblotting with anti-Atg13 antibody (upper). A non-specific band was used as a protein loading control (bottom).

Pib2 is a protein comprising 635 amino acids that specify two well-conserved motifs, a FYVE domain and a tail motif, and five weakly conserved motifs that are designated A-E motifs (Fig 6A) [21]. We next examined which domain of Pib2 is responsible for binding to TORC1 by expressing truncated versions of Pib2 fused to GFP in Δ*pib2* cells. Association with Tor1 was subsequently assessed by immunoprecipitation using buffers containing glutamine. All Pib2 mutants containing Motif E interacted with Tor1, although the amount of Tor1 co-precipitated with Pib2^304-635^ was less than the others (Fig 6B, S6 Fig). On the other hand, Pib2^440-635^, which lacks motif E, almost completely lost the ability to associate with Tor1, suggesting that motif E is required for the interaction between Pib2 and TORC1 (Fig 6B, S6 Fig).

**Fig 7:**
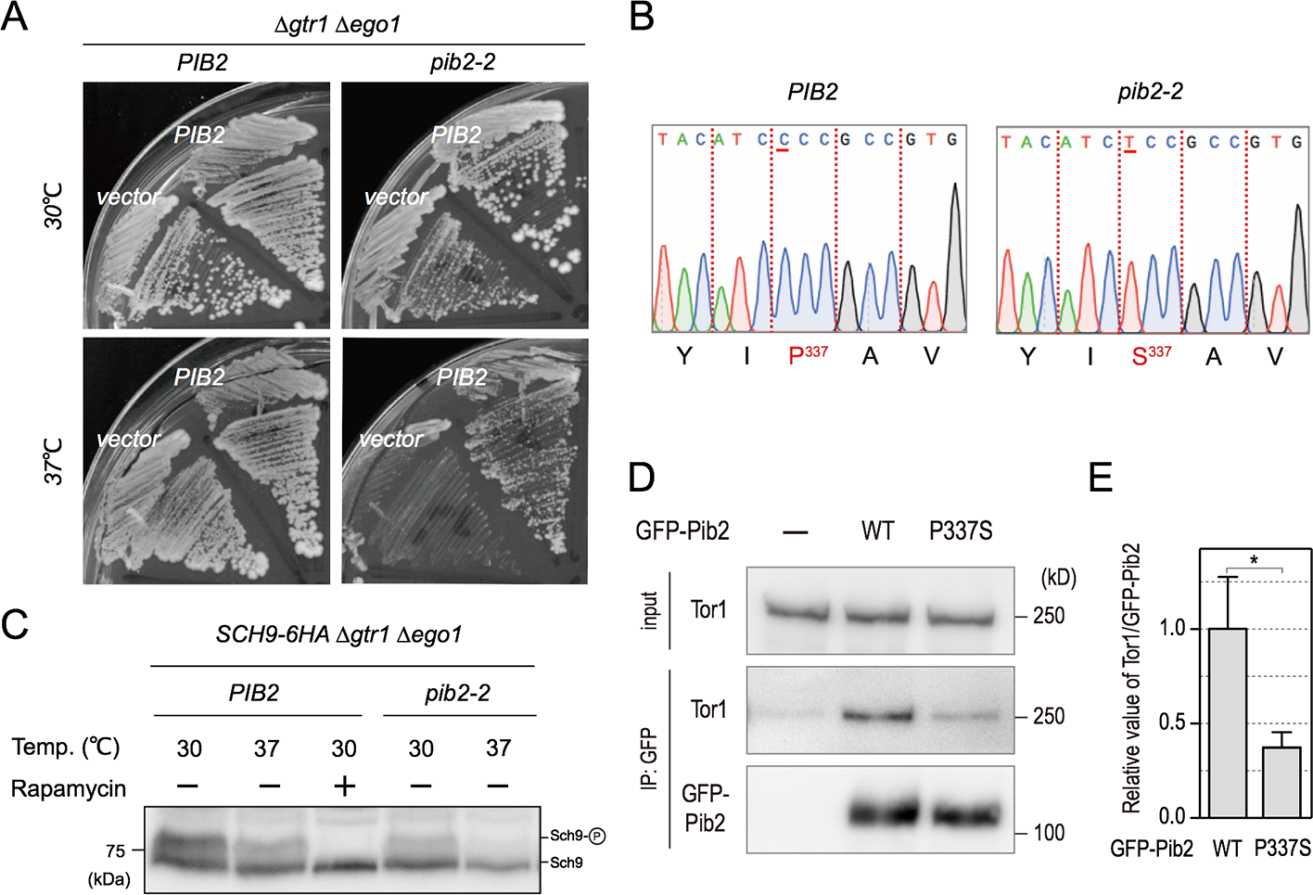
Proline residue 337 in motif E is required for the activation of TORC1 activity. (A) *pib2-2* Δ*gtr1* Δ*ego1* cells (YAY2543) transformed with a CEN plasmid encoding *PIB2* or an empty (control) plasmid were tested for growth as indicated. (B) Sequencing revealed a single nucleotide change, resulting in a proline to serine alteration at the 337th residue in motif E. (C) Δ*gtr1* Δ*ego1* cells with either *PIB2* or *pib2-2* expressing Sch9-6HA (YAY2592, YAY2607) were cultured at 30°C or 37° for 2 hours. For the analysis of Sch9 phosphorylation, lysates were treated with NTCB and subjected to western blotting using an anti-HA. (D) Δ*pib2* cells expressing GFP-Pib2^WT^ (YAY2731) and GFP-Pib2^P337S^ (YAY2732) were grown in YPD. Extracts were prepared and immunoprecipitated by GFP-Trap. Whole-cell extract (top, “input”) and precipitated proteins (bottom) were analyzed by western blotting using anti-Tor1 and anti-GFP antibodies. (E) Quantification of the ratio of Tor1/GFP-Pib2 in (D). Data are shown as mean ± SD (n = 3) as calculated by the student’s *t*-test. *p < 0.05.

We further investigated this possibility by expressing the truncated Pib2 variants in Δ*pib2* cells and assessing their effect on TORC1 activation and rapamycin sensitivity. Two mutants (Pib2^165-635^ and Pib2^304-635^), which interact with Tor1, restored the ability of glutamine to activate TORC1 and rendered cells resistant to rapamycin (Fig 6C and 6D). As reported in a recent paper by Michel et al., cells expressing N-terminally truncated variants of Pib2 were more resistant to rapamycin than wild-type cells, and the extent of this resistance was comparable to that of cells expressing a Pib2^50-635^ truncation mutant, indicating that only the first 50 N-terminal residues of Pib2 may have a negative impact on TORC1 activity (Fig 6C) [23]. In contrast, two other variants were not able to rectify TORC1 activation in Δ*pib2* cells (Fig 6C and 6D). The first of these, Pib2^440-635^, lacks motif E and does not interact with Tor1. The second, Pib2^1-620^ includes motif E but lacks the tail motif of the protein. This is consistent with previous data suggesting that removal of the tail motif completely diminishes complementation in Δ*pib2* cells [21]. Furthermore, we isolated *pib2-2*, in which we identified a proline to serine mutation at the 337th residue of Pib2, which is within motif E of the protein. This point mutation confers temperature sensitivity on Δ*gtr1* Δ*ego1* cells (Fig 7A and 7B). The 337th proline residue is highly conserved among diverse fungal species, suggesting its functional importance in the Pib2 protein.

TORC1 activity in *pib2-2* cells at restrictive temperature was examined by monitoring phosphorylation of Sch9 (Fig 7C). Sch9 phosphorylation was nearly completely abolished when considering a *pib2-2* mutant in a Δ*gtr1* Δ*ego1* background at 37°C, reproducing our findings following rapamycin treatment (Fig 7C). To confirm the reliability of this approach, we also assessed the stability of the GFP-Pib2^P337S^ proteins at 37°C. Western blot analysis revealed that steady levels of GFP-Pib2^P337S^ and GFP-Pib2^wt^ are comparable (S7A and S7B Fig). Moreover, the interaction between Pib2^P337S^ and Tor1 is compromised even at 4°C, as shown in Fig 7D and 7E, demonstrating that the failure of GFP-Pib2^P337S^ protein to interact with Tor1 leads to the inactivation of TORC1 in Δ*gtr1* Δ*ego1* cells. Taken together, these data strongly suggest that motif E of Pib2 is required for glutamine-induced TORC1 activation through its interaction with TORC1.

### Glutamine binds directly to the Pib2 complex

**Fig 8:**
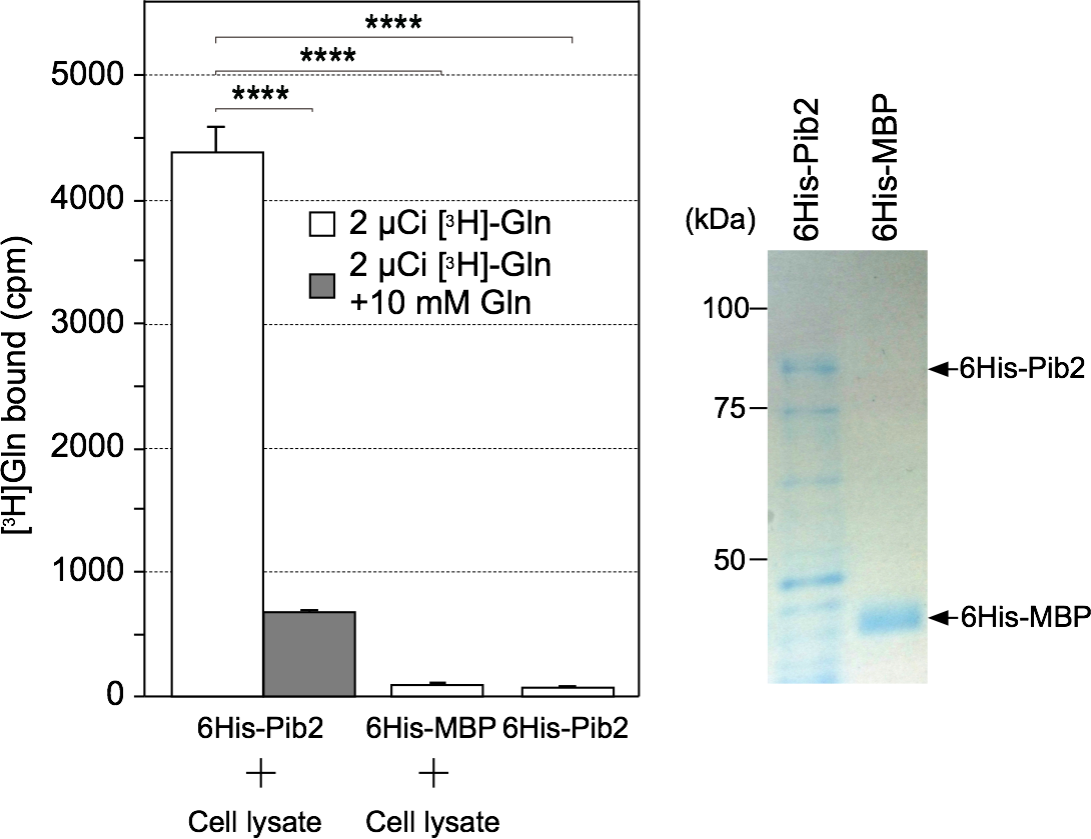
The Pib2 complex binds glutamine. Recombinant 6His-Pib2 or 6His-MBP protein on Ni-NTA agarose was incubated without or with cell lysates of Δ*pib2* cells (YKOL4391) for 60 min at 4°C. After washing, the [^3^H]l-glutamine-binding assay was performed as described in Materials and Methods. Unlabeled glutamine was added where indicated. Statistical data are shown as Mean ± SE of three independent experiments. ****p < 0.0001, Student’s *t*-test. The purified proteins were also separated by SDS-PAGE and visualized by Coomassie staining (right panel).

Based on the above results, we hypothesized that Pib2 itself is a glutamine sensor. To test this, we first developed a glutamine equilibrium binding assay using the glutamine-binding protein (GlnBP) from *Escherichia coli* as a positive control (S8A Fig) [36]. However, we were not able to detect any significant binding of Pib2 recombinant proteins purified from *E*. *coli* with glutamine (Fig 8). We therefore tested if a yet-unidentified Pib2-binding protein is able to sense glutamine directly or facilitate the binding of Pib2 to glutamine. We incubated purified 6His-Pib2 or 6His-MBP immobilized on Ni-beads with cell extracts prepared from Δ*pib2* cells, followed by extensive washing of beads to elute unbound protein. Beads were then incubated in the presence of radiolabeled glutamine, and the level of glutamine binding was quantified after washing of the beads [14]. When incubated with cell extracts, we found that radioactive glutamine binds 6His-Pib2 but not 6His-MBP in a manner that is almost fully competitively inhibited when excess unlabeled glutamine is supplied (Fig 8). In contrast to glutamine, tritium-labeled leucine binds 6His-Sestrin2 but not 6His-Pib2 nor 6His-MBP, in spite of the treatment of yeast cell extracts, further underscoring the specificity of Pib2 binding with glutamine (S8B Fig). Thus, we conclude that Pib2 acts as a part of a putative glutamine sensor in cooperation with a yet unidentified protein that is able to interact with Pib2.

## Discussion

In this study, we have addressed the question of how the dual Pib2- and Gtr-dependent pathways are dynamically engaged in TORC1 activation in response to specific amino acids. The most obvious and important question concerns what signal these pathways respond to. We recapitulated the pioneering results of Stracka *et al*., who showed that TORC1 activity exhibits different responses to glutamine and other amino acids (as exemplified by leucine) and proposed the existence of a Gtr-independent mechanism of TORC1 activation by glutamine [19]. More recent studies have suggested that Pib2 is involved in the glutamine-dependent activation of TORC1 independently of the Gtr/Ego system [21–23]. This notion was confirmed and extended further by the following findings presented in this work. First, through the use of a double depletion experiment, we found that in the absence of both Gtr1 and Pib2 TORC1 activity is completely blocked (Fig 1D). This finding is underscored by our localization studies, which indicate that the double depletion of Gtr1 and Pib2 causes a complete defect in TORC1 localization to the vacuole (Fig 4D). Together, these data therefore provide a strong indication that the Gtr/Ego system and Pib2 function as the sole regulators of TORC1 activation, although we cannot exclude the possibility that other proteins may be involved in TORC1 regulation under specific conditions other than nitrogen starvation. Second, we comprehensively demonstrate that while both Pib2 and Gtr1 interact with TORC1, they do not interact with each other (Fig 2A–2C, S2B and S2C Fig). This direct evidence for the existence of distinct TORC1 pools explains the specialized roles of the two pathways in amino acid response. Third, our protein analyses of Pib2 functional domains provide conclusive evidence that the E domain (Fig 6B, S6 Fig), and in particular the 337^th^ proline residue (Fig 7D and 7E), is essential for binding with TORC1, and that interaction between the E domain and TORC1 results in TORC1 activation and thereby rapamycin resistance, which is an important characterization of the Pib2 protein (Fig 6C and 6D, Fig 7C). Forth, we very clearly show that Pib2-mediated TORC1 regulation is dependent on the concentration of glutamine by immunoprecipitation studies (Fig 5A–5C). This result draws a direct link between glutamine availability and Pib2 function. Finally, we provide evidence that Pib2 plays a role as a part of a glutamine sensor, although the molecular context of this function remains to be established (Fig 8).

In sharp contrast to ours and the previous observation by Stracka *et al*., Varlakhanova *et al*. claim that Gtr/Ego and Pib2 work together to activate TORC1, rather than independently, based on the fact that both Pib2 and Gtr/Ego are required for glutamine and leucine-induced TORC1 activation [37]. In this paper, the authors use the Δ*gtr1* Δ*gtr2* double knockout strain as a Gtr/Ego mutant instead of the Δ*gtr1* single knockout strain, as we used in our analyses. The Δ*gtr1* Δ*gtr2* double knockout may be a reason for this discrepancy. Stracka *et al*. previously noted that Δ*gtr2* cell growth is poor in comparison to Δ*gtr1* cells due to a more prominent role of Gtr2 in permease sorting [19]. This is consistent with the strong phenotype of the Δ*gtr1* Δ*gtr2* double knockout strain in terms of the amino-acid response. Furthermore, there are two remarkable differences between our experiments and the approach of Varlakhanova *et al*. First, their parental strain is auxotrophic for amino acids (W303), but we and Stracka *et al*. used a prototrophic strain (FY3). By employing a prototrophic strain, we were able to avoid the potential confounding effects arising from the requirement of additional amino acids for growth. Second, we assessed the phosphorylation state of Sch9 to monitor changes in TORC1 activity. Sch9, which is a direct target of TORC1 phosphorylation, is a well-established and widely employed assay in the TORC1 field [6]. On the other hand, Varlakhanova *et al*. monitored TORC1 activity based on the phosphorylation state of the 40S ribosomal subunit Rps6, which is not a direct target of TORC1, at residues Ser232/Ser233. Recently, it has been reported that TORC1, via Ypk3, regulates Ser-232 and Ser-233 phosphorylation, whereas TORC2 also regulates Ser-232 phosphorylation via Ypk1 and Ypk2 [38]. Thus, the interrogation of Rps6 phosphorylation alone as a measure of TORC1 activity must be interpreted with caution. In addition to the different genetic backgrounds used in these experiments, these additional technical factors may contribute to the observed discrepancies. Although beyond the scope of this present study, it would be interesting to further investigate the cause of these contradictory findings in a future study.

The vacuolar localization of Pib2 is only partially dependent on its FYVE domain, which is responsible for the association of proteins with PtdIns3P [21], suggesting that another mechanism mediated by PtdIns3P may exist for its vacuolar localization (Fig 3B). It is unlikely that residual Pib2 observed on the vacuole membrane is caused by binding to Gtr/Ego–anchored TORC1, as Tor1 is localized to the vacuole in Δ*vps34* cells [22]. Despite only a partial localization defect, mutations in the FYVE domain lead to the impairment of TORC1 activity, indicative of the direct involvement of PtdIns3P in Pib2-mediated TORC1 activation (Fig 3C). This enabled us to assess the significance of the vacuolar localization of Pib2 in TORC1 activation. Forced tethering of the Pib2 FYVE mutants on the vacuolar membrane bypassed the requirement for the FYVE domain (Fig 3B and 3C). Although PtdIns3P metabolism is implicated in mTORC1 regulation in mammalian cells [39–41], and the yeast Δ*vps34* mutant is also rapamycin-sensitive [42], our data now demonstrate that the vacuolar localization of Pib2 alone is required for its function in TORC1 activation and that the binding of the FYVE domain to PtdIns3P plays no role other than to determine Pib2 localization.

Our immunoprecipitation results provide further evidence that Pib2 and Gtr/Ego are present in distinct TORC1-containing complexes (Fig 2A–2C, S2B and S2C Fig). Both Pib2 and Gtr/Ego co-localize on the vacuolar membrane and in associated puncta at the same time, migrating comparably between these localizations in response to amino acid supplementation (Fig 4B). Moreover, the Gtr/Ego system to a degree modulates the vacuolar membrane localization of Pib2 (S4C and S4D Fig). Therefore, we cannot rule out the possibility that the Pib2- and Gtr-containing TORC1 complexes are associated with each other in a weak manner that is not detectable by conventional immunoprecipitation methods. Like mTORC1, yeast TORC1 may form a homodimer [43], and if so, it could contribute to potential hetero-association between Pib2-Gtr complexes and homo-association of the Pib2 complex. Such a higher-order relationship between the Pib2 and Gtr pathways may underlie the simultaneous requirement for Pib2 and Gtr in the response to leucine. In fact, a genome-wide *in vivo* screen for protein-protein interactions revealed that Pib2 is localized in close proximity to Gtr1 and Gtr2 [44]. However, this system is based on protein-fragment complementation. A problem with the approach as used in this investigation of Pib2 is that an mDHFR fragment was fused to the C-terminal region of Pib2. We and other groups have observed that the introduction of a C-terminal tagging sequence to the *PIB2* gene impairs the function of the encoded Pib2 protein, although it is not yet clear why. The physiological significance of this reported interaction therefore remains dubious. Given that Pib2, but not PtdIns3P, forms puncta in the absence of the Gtr system (S4C–S4E Fig), it is possible that multiple Pib2-containing TORC1 complexes could undergo some type of self-oligomerization (Fig 4C). During revision, Prouteau *et al*. reported that in Δ*gtr1* Δ*gtr2* cells, TORC1 oligomerizes into a higher-level helical assembly that has been named a TOROID [45]. Gtr1 and Gtr2 play a role in the disassembly of the TOROID in response to nutrients, although the mechanistic details remain uncharacterized. We speculate that vacuole-associated GFP-Pib2 puncta in Δ*gtr1* cells could be Pib2 proteins that have become stuck in TOROIDs. This is consistent with the fact that we were unable to immunoprecipitate GFP-Pib2 under nitrogen starvation conditions in which GFP-Tor1 exhibits punctate structures (Fig 5A). In this scenario, a Gtr/Ego-independent localization of Pib2 on the vacuolar membrane could be possible, but currently it is difficult to address this problem experimentally in the absence of mechanistic details of Gtr1 and Gtr2 function in TOROID formation.

The question of whether amino acid sensors exist remains controversial in yeast, although in mammals Sestrin and CASTOR have been identified as leucine and arginine sensor proteins, respectively [14,15]. In this work, we demonstrated the following: (1) l-glutamine but neither d-glutamine nor l-leucine facilitates the interaction between Pib2 and TORC1 *in vivo* and *in vitro* (Fig 5A–5C and 5E, S5C Fig), and (2) glutamine directly binds the Pib2 complex but not Pib2 alone (Fig 8). These data strongly suggest that Pib2 is a part of a glutamine sensor. We propose a model describing how a glutamine signal is transduced through the vacuolar protein Pib2. Without glutamine, TORC1 cannot associate with the vacuole. On the other hand, the Pib2 complex, which is tethered to the vacuolar membrane through the interaction between its FYVE domain and PtdIns3P generated by Vps34, binds to glutamine directly in the presence of cytoplasmic glutamine. The binding of the Pib2 complex to glutamine facilitates Pib2–TORC1 complex formation, allowing TORC1 to associate with the vacuolar surface, where it is subsequently activated through an unknown mechanism. We attempted to identify glutamine sensor molecules that interact with Pib2 by mass spectrometry, and while all components of TORC1 were identified using this approach (Fig 2C, Fig 5C), our search for glutamine-sensing molecules yielded no obvious candidates. Identification of such a molecule will provide a deeper understanding of the molecular details of glutamine’s function in TORC1 activation, as well as Rag-independent mTORC1 activation by glutamine in mammals [46].

## Materials and methods

### Yeast strains, media

The yeast strains used in this study are listed in S1 Table. Yeast cells were grown in YPD (1% yeast extract (BD Biosciences); 2% peptone (BD Biosciences); 2% glucose (Wako)), SCD (0.17% yeast nitrogen base without amino acids and ammonium sulfate (BD Biosciences), 0.5% (NH_4_)_2_SO_4_ (nacalai tesque), 0.5% casamino acid (BD Biosciences), 2% glucose (Wako)), or YMM. This detailed composition of YMM is as follows (for 1 liter): 3.0 g of KH_2_PO_4_ (Wako), 0.5 g of MgSO_4_•7H_2_O (Wako), 5.0 g of K_2_SO_4_ (Wako), 15 mg of EDTA (Sigma), 4.5 mg of ZnSO_4_•7H_2_O (Wako), 0.3 mg of CoCl_2_•6H_2_O (Wako), 1.0 mg of MnCl_2_•4H_2_O (Wako), 0.3 mg of CuSO_4_•5H_2_O (Wako), 4.5 mg of CaCl_2_•2H_2_O (Wako), 3.0 mg of FeSO_4_•7H_2_O (Wako), 0.4 mg of NaMoO_4_•2H_2_O (Wako), 1.0 mg of H_3_BO_3_ (Wako), 0.1 mg of KI (nacalai tesque), 0.05 mg of biotin (TCI), 1 mg of calcium pantothenate (nacalai tesque), 1 mg of nicotinic acid (nacalai tesque), 25 mg of inositol (nacalai tesque), 1 mg of pyridoxine (nacalai tesque), 0.2 mg of *p*-aminobenzoic acid (nacalai tesque), 1 mg of thiamine (Wako), 0.002 mg of Folic acid (nacalai tesque), 0.2mg of riboflavin (nacalai tesque), 2% glucose (Wako); volume adjusted to 1.0 liter with 10 mM potassium hydrogen phthalate•H_2_O (pH 5.0) (Wako) [25]. Ammonium sulfate (final conc. 0.5 g/liter) (nacalai tesque), the auxotrophic supplement uracil (final conc. 20 mg /liter) (Wako), or various amounts of l-glutamine (Wako), d-glutamine (Wako) or l-leucine (Sigma) were added where indicated. Rapamycin (53123-88-8; LKT Laboratories) in stock solution (1 mg/ml ethanol and Triton X-100 (Wako) at a ratio of 9:1 (v/v)) was added to YPD to achieve a final concentration of 0.2 μg/ml. Cells were grown in SCD media, and genetic depletion was initiated by addition of doxycycline to growth media at a final concentration 4 μg/ml. For non-depleted controls, the same volume of ethanol (solvent) was added.

### Microscopy

Cells were grown in SCD or YMM medium containing ammonium sulfate as the sole nitrogen source, and then shifted to nitrogen-free YMM for 30 minutes, after which the indicated amino acids were added. Cells were collected by centrifugation (600 x g, 2 min) and subjected to microscopy. The cells were observed on a Leica AF6500 fluorescence imaging system (Leica Microsystems) mounted on a DMI6000B microscope (HCX PL APO 100/1.40–0.70 oil-immersion objective lens, xenon lamp (Leica Microsystems)) under the control of the LAS-AF software (Leica Microsystems). For time-lapse imaging, the cells were grown in YMM medium containing ammonium sulfate as the sole nitrogen source, and then shifted to nitrogen-free YMM on a glass bottom dish (Matsunami Glass) mounted with 2 mg/ml of concanavalin A (Sigma). Cells were then subjected to time-lapse imaging after addition of glutamine (final concentration of 0.5 mg/ml). Images were recorded using a DeltaVision Personal system (Applied Precision) mounted on a IX71 microscope (UPlanSApo 100x/1.40 oil-immersion objective lens, LED lamp (OLYMPUS)). ImageJ software (National Institutes of Health) was used to process and produce merged images.

### NTCB treatment for Sch9

NTCB treatments were performed as previously reported with slight modifications [6]. Briefly, 8 OD units of cells were treated with 6% trichloroacetic acid (Wako) for at least 15 minutes on ice, washed twice with ice-cold acetone, and dried using a SpeedVac. The pellets were re-dissolved in 200 μl of urea buffer (50 mM Tris-Cl (pH 7.5) (Sigma), 5 mM EDTA (Wako), 6 M urea (Wako), 1% SDS (nacalai tesque), Complete EDTA-free protease inhibitor cocktail (Roche), 1 mM PMSF (Wako), 1 μM microcystin-LR (Wako), and PhosSTOP (Roche)) and lysed with the FastPrep instrument (MP-Biomedicals) and 0.6-mm-diameter zirconia beads (Biomedical Science). After centrifugation at 20,000 × *g* for 10 min at 4°C, 100 μl of supernatant were transferred to a new 1.5 ml reaction tube. The lysates were mixed with 30 μl of 1 M 2-(cyclohexylamino) ethanesulfonic acid (pH 10.5) (Wako) and 20 μl of 7.5 M NTCB (Sigma) and incubated overnight at room temperature. Each sample was mixed with 50 μl of 4×loading buffer (800 mM Tris-Cl (pH 6.8) (Sigma), 6% SDS (nacalai tesque), 400 mM dithiothreitol (Wako), 8 M urea (Wako), 0.04% bromophenol blue(Sigma)) and subjected to SDS-PAGE and western blot analysis.

### Antibodies

Anti-protein A (P-3775; Sigma-Aldrich), anti-GFP (11814460001; Roche), anti-Tor1 (sc-11900; Santa Cruz Biotechnology), anti-PGK (459250; Thermo Fisher Scientific), rabbit anti-Goat IgG HRP (anti-TAP) (ab6741; abcam), goat anti-mouse IgG, human ads-HRP (1030–05; Southern Bio Tech), anti-rabbit IgG HRP-linked antibody (7074S; Cell signaling), anti-Atg13 (a gift from Dr. Yoshinori Ohsumi, Tokyo Institute of Technology), and anti-HA (901501; BioLegend) antibodies were used for western blotting.

### Immunoprecipitation experiments

Cells were resuspended in TAP-A buffer (50 mM Tris-HCl (pH 8.0) (Wako), 150 mM NaCl (Wako), 10% glycerol (Wako), 1 mM DTT (Wako), 1 mM EDTA (Sigma) supplemented with Complete EDTA-free protease inhibitor cocktail (Roche), 1 mM PMSF (Wako), 1 μM microcystin-LR (Wako) and PhosSTOP (Roche), and lysed using a FastPrep instrument (MP-Biomedicals) and zirconia beads. After lysis, cell lysates were incubated for 10 min at 4°C following addition of Triton X-100 (0.2% final concentration) (Wako), and then clarified by centrifugation at 20,000 × *g* for 10 min at 4°C. Gtr1-TAP proteins were precipitated with magnetic beads covalently coupled to rabbit IgG (Dynabeads M-270 Epoxy beads: Invitrogen). GFP-tagged Pib2 proteins were precipitated with magnetic GFP-Trap-M beads (Chromotek). The beads were washed three times with TAP-A buffer containing 0.2% of Triton X-100. In S2B Fig, Rag buffer (40 mM Na-HEPES (pH 7.4) (DOJINDO), 5 mM MgCl_2_ (WAKO), 150 mM NaCl_2_ (WAKO)) was used instead of TAP-A buffer. In Fig 5 and S5 Fig, all buffers were added with the indicated final concentration of l-glutamine, d-glutamine or l-leucine. Bound proteins were eluted in SDS sample buffer by heating for 5 min at 95°C. Proteins were resolved by SDS-PAGE and analyzed by standard western blotting techniques.

### Phosphatase treatment

*GFP-PIB2* cells grown in YPDA without or with rapamycin were lysed and GFP-PIB2 was immunoprecipitated as described in the previous section. Reactions both with and without lambda phosphatase (New England Biolabs) were set up and incubated at 30°C for 30 min. Prior to removal from the beads, the beads were washed once with lysis buffer. HU buffer was then added to each sample, which were then incubated at 65°C for 15 min to elute the bound proteins. Phos-tag (Wako) was used to detect the mobility shift by phosphorylation.

### LC-MS/MS analysis

Cells expressing TAP-tagged proteins were resuspended in Lysis150 buffer (50 mM Tris-HCl pH 8, 150 mM NaCl, 10% glycerol, 1 mM dithiothreitol (DTT), 0.2% Triton X-100) supplemented with 1 mM PMSF, Complete EDTA-free protease inhibitor cocktail (Roche), and PhosSTOP (Roche), and were then lysed using a glass bead homogenizer. TAP-tagged proteins were immunoprecipitated using rabbit IgG (Sigma) conjugated to M270 epoxy Dynabeads. Bound proteins were eluted by Laemmli buffer, followed by SDS-PAGE. The proteins were excised from each gel, destained and digested in the gels with 12.5 ng/μl trypsin (Wako) in 50 mM ammonium bicarbonate overnight at 37°C. The peptides were desalted with 3 M Empore C18 Solid Phase Extraction Disks (Sigma). NanoLC-MS/MS analysis was conducted using a Q Exacitve hybrid quadrupole-orbitrap mass spectrometer (Thermo Fisher Scientific), with Xcalibur software, and coupled to an EASY-nLC 1000 (Thermo Fisher Scientific). The data were processed, searched and quantified using Proteome Discoverer (version 2.1.0.81, Thermo Fisher Scientific), employing the *S*. *cerevisiae* UniProt database (version Feb. 21, 2016) containing 6749 entries. The search parameters were as follows: trypsin digestion with two missed cleavage permitted; variable modifications, protein N-terminal acetylation, oxidation of methionine, propionamidation of cysteine and phosphorylation of serine, threonine and tyrosine; peptide charge (2+, 3+ and 4+); peptide mass tolerance for MS data, ±10 p.p.m.; and fragment mass tolerance, ±0.02 Da. The abundance of each protein was quantified by the exponentially modified Protein Abundance Index (emPAI), which provides an estimate of absolute abundance of proteins by quantitating the number of peptides identified by MS [35]. To compare results from multiple purifications, relative abundances of proteins defined as emPAI_prey_/emPAI_bait_ were calculated.

### Amino acid binding assay

6His-fused Pib2, 6His-MBP, 6His-GlnBP-MBP and 6His-Sestrin2 proteins were expressed in *E*. *coli* Rosetta2 (DE3) by adding 0.25 mM IPTG for 3 h at 30°C and purified using Ni-NTA beads (QIAGEN) according to the manufacture’s protocol. A portion of the purified proteins were visualized by CBB to confirm purity. Δ*pib2* cells were resuspended in lysis buffer (50 mM Tris-Cl pH 8, 150 mM NaCl, 10% glycerol, 1 mM dithiothreitol (DTT), 0.2% Triton X-100, 1 mM EDTA) supplemented with 1 mM PMSF, Complete protease inhibitor cocktail EDTA free (Roche), PhoSTOP (Roche) and 1 μM microcysteine-LR and were lysed with a glass bead homogenizer. The cleared cell extract was then incubated with Ni-NTA beads containing 6His-Pib2 or 6His-MBP for 60 min at 4°C. The beads were washed thoroughly three times in lysis buffer and resuspended in 200μl of the lysis buffer. The beads were incubated with 2 μCi of l-[3,4-^3^H(N)]-glutamine or l-[4,5-^3^H(N)]-leucine on ice for 30 min (with shaking every five minutes) in the presence or absence of 10 mM unlabeled glutamine or leucine. After incubation, the beads were spun down (3,000g for 1 min), and washed three times with lysis buffer. The beads were then resuspended in 900 μl lysis buffer and equally split into three separate scintillation tubes containing 5 ml of Ultima Gold (PerkinElmer) for quantification by a scintillation counter (PerkinElmer).

### Generation of conditional lethal *pib2* alleles

The *pib2-2* allele was obtained by random in vitro mutagenesis of *PIB2-kanMX4* with a primer upstream of the promoter and one downstream of the antibiotics marker. The Mutagenized DNA of *PIB2* was integrated by homologs recombination into *PIB2* locus in the Δ*gtr1* Δ*ego1* strain (YAY2531) and cells were screened for temperature sensitive alleles that could grow on YPD plates supplemented with G418 at 30°C but not at 37°C.

### Statistical analyses

Significance of differences was determined using an unpaired two-tailed Student’s *t* test or Mann-Whitney *U*-test. ns stands for “not significant”.

### Resource availability

All materials will be made freely available upon request.

## Supporting information

S1 Fig Related to Fig 1: Pib2 is a core component of the glutamine-responsive pathway for TORC1 activation. (A) Spore analysis of Δ*pib2/*Δ*gtr1* (HUY29/YKOL6522) and Δ*pib2/*Δ*ego1* (HUY29/YKOL5078) cells. Numbers indicate cells that developed from spores of one complete tetrad. Genotypes were determined by replica plating on media containing either G418 or zeocin after 2 days of growth at 30°C. (B, C and D) Cells of the indicated genotypes (Wild type: SKY384, Δ*gtr1*: HUY33, Δ*pib2*: HUY34) were grown in YMM medium supplemented with ammonium sulfate, and then shifted to nitrogen-free YMM. Thirty minutes after the shift, glutamine (B and C) or leucine (D) were added at 3 mM. Phosphorylation of Sch9 (B and D) or Atg13 (C) was monitored at each time point by immunoblotting with the indicated antibodies (left panels). For the analysis of Sch9 phosphorylation, lysates were treated with NTCB and subjected to western blotting using an anti-HA antibody. Relative quantification of Sch9 or Atg13 phosphorylation is shown as the mean ± SE (n = 3 in B and D, n = 2 in C) (right panels). (E) Growth curve of Cells of the indicated genotypes (Wild type: SKY384, Δ*gtr1*: HUY33, Δ*pib2*: HUY34) in YMM medium supplemented with 3 mM leucine. Statistical data are shown as Mean ± SE of two independent experiments.
(TIF)

S2 Fig Related to Fig 2: Pib2 constitutes a complex with TORC1 distinct from the Gtr1-containing complex. (A) Cells of the indicated genotypes were serially diluted 10-fold and spotted on YPD plates with 0.2 μg/ml rapamycin, and then grown at 30°C for 3 days. (B) Cells expressing the indicated tagged proteins were grown in SCD and analyzed as described in Fig 2A (*GTR1-TAP GFP-PIB2*: HUY57 with pRS316-*GTR1-TAP*, *GTR1-TAP GFP*: HUY77 with pRS316-*GTR1-TAP*, *TAP GFP-PIB2*: HUY58 with pRS316-*GTR1*). (C) Quantification of the ratio of IP/input in (B). Mean ± SD (n = 3). **p < 0.01, ****p < 0.0001, Student’s *t*-test.
(TIF)

S3 Fig Related to Fig 3: Related to Fig 3: The FYVE-mutant proteins of Pib2 are expressed at similar levels to the wild-type protein. (A) Cells were cultured as described in Fig 3B. Cell extracts were prepared, then analyzed by immunoblotting with anti-GFP and anti-Pgk1 antibodies. (B) Quantification of the ratio of GFP-Pib2/Pgk1 in (A). Mean ± SD (n = 3). Significance was calculated using the student’s *t*-test.
(TIF)

S4 Fig Related to Fig 4: GFP-Tor1 and GFP-Pib2 exhibit punctate structures under nitrogen starvation conditions. (A) Quantification of cells with vacuolar membrane associated puncta from 100–200 cells in Fig 4A. Mean ± SE (n = 4). *p < 0.05, Mann-Whitney *U*-test. (B) Quantification of cells with the vacuolar membrane associated puncta from 100–200 cells in Fig 4C. Mean ± SE (n = 4). *p < 0.05, Mann-Whitney *U*-test. (C) Cells expressing GFP-Pib2 in Δ*gtr1* (HUY59) were cultured and analyzed by fluorescence microscopy as in Fig 3A. Statistical data are shown as mean ± SD from 100–200 cells of four independent experiments. (D) Quantification of cells with the vacuolar membrane associated puncta from 100–200 cells in (C). Mean ± SE (n = 4). *p < 0.05, Mann-Whitney *U*-test. (E) Wild type (BY4741) or Δ*gtr1* (YKOL6522) cells harboring the *GFP-FYVE* plasmid (pRS425-*GFP-FYVE*) were cultured and analyzed by fluorescence microscopy, as in Fig 3A.
(TIF)

S5 Fig Related to Fig 5: l-glutamine, but not l-leucine, reinforces the Tor1-Pib2 interaction *in vitro*. (A) Quantification of the ratio of Tor1/GFP-Pib2 in Fig 5A. Mean ± SD (n = 3). *p < 0.05, ***p < 0.001, Student’s *t*-test. (B) Cells grown with or without rapamycin were collected after 3 h. Immunoprecipitated GFP-Pib2 were incubated as indicated and analyzed by immunoblotting with anti-GFP antibody. (C) Quantification of the ratio of Tor1/GFP-Pib2 in Fig 4B. Mean ± SE (n = 4). *p < 0.05, Student’s *t*-test. (D) Cells expressing GFP-Pib2 (HUY45) were grown in YPD. Different concentrations of either l-glutamine or l-leucine were added to all buffers used in the experiment, and cell lysates were prepared and analyzed as in Fig 5A. (E) Quantification of the ratio of Tor1/GFP-Pib2 in (D). Mean ± SD (n = 3). Student’s *t*-test. (F) Quantification of the ratio of Tor1/GFP-Pib2 in Fig 5E. Mean ± SD (n = 3). **p < 0.01, Student’s *t*-test.
(TIF)

S6 Fig Related to Fig 6: Motif E of Pib2 is required for the interaction with Tor1. Quantification of the ratio of Tor1/GFP-Pib2 in Fig 6B. Mean ± SE (n = 3). *p < 0.05, **p < 0.01, Student’s *t*-test.
(TIF)

S7 Fig Related to Fig 7: Protein levels of GFP-Pib2^ts^ and GFP-Pib2^wt^ are comparable at 37°C. (A) Δ*pib2* cells expressing GFP-Pib2^WT^ (YAY2731) and GFP-Pib2^P337S^ (YAY2732) were grown at 30°C and then harvested. The cells were resuspended in fresh pre-warmed medium and incubated at 37°C for 1 or 3 h. Lysates were subjected to western blotting using anti-GFP and anti-Pgk1 antibodies. (B) Quantification of the ratio of GFP-Pib2/Pgk1 in (A). Mean ± SD (n = 3). Student’s *t*-test.
(TIF)

S8 Fig Related to Fig 8: The Pib2 complex does not bind leucine. (A) Recombinant 6His-GlnBP or 6His-MBP protein on Ni-NTA agarose was incubated with [^3^H]l-glutamine for 30 min at 4°C. After washing, the [^3^H]l-glutamine-binding assay was performed as described in Materials and Methods. Unlabeled glutamine was added where indicated. Statistical data are shown as mean ± SE of three independent experiments. **p < 0.01, Student’s *t*-test. The purified proteins were separated on SDS-PAGE and visualized by Coomassie staining (right panel). (B) Recombinant 6His-Pib2, 6His-MBP or 6His-Sestrin2 protein on Ni-NTA agarose was incubated without or with cell lysates of Δ*pib2* cell (YKOL4391) for 60 min at 4°C. After washing, the [^3^H]l-leucine-binding assay was performed as described in Materials and Methods. Unlabeled leucine was added where indicated. Statistical data are shown as Mean ± SE of three independent experiments. ****p < 0.0001, ***p < 0.001, Student’s *t*-test. The purified proteins were separated on SDS-PAGE and visualized by Coomassie staining (right panel).
(TIF)

S1 Video TORC1 is dynamically translocated in response to glutamine. GFP-Tor1 (SKY222) cells were grown in SCD supplemented with uracil and adenine, and then shifted to nitrogen-free YMM for 30 minutes on a glass bottom dish coated with 2 mg/ml of concanavalin A. Cells were then subjected to time-lapse imaging after the addition of glutamine (final concentration of 0.5 mg/ml) at 1 min intervals. Frames are displayed at 4 frames/second. Scale bar, 5 μm.
(AVI)

S1 Table Yeast and *E*. *coli* strains used in this study. (PDF)

S2 Table List of proteins identified by LC-MS/MS in Fig 2C and Fig 5C. (XLSX)

S3 Table Numerical data underlying graphs. (XLSX)
