# Supplementary figures and images for: Gtr/Ego-independent TORC1 activation is achieved through a glutamine-sensitive interaction with Pib2 on the vacuolar membrane
Source: PLoS Genet. 2018 Apr 26;14(4):e1007334. doi: 10.1371/journal.pgen.1007334 (PMC5919408; doi:10.1371/journal.pgen.1007334)

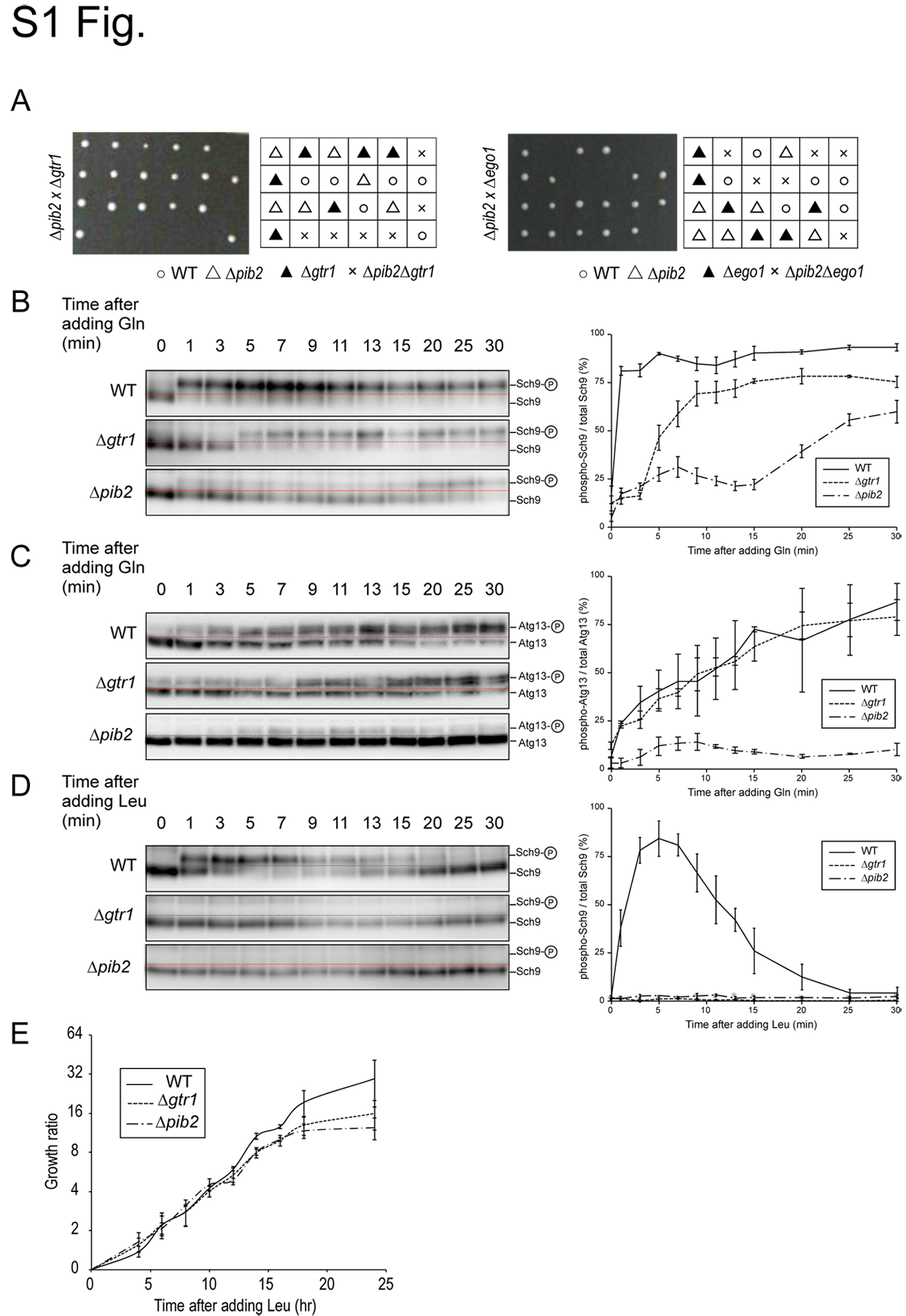

Supplement: S1 Fig — (A) Spore analysis of Δpib2/Δgtr1 (HUY29/YKOL6522) and Δpib2/Δego1 (HUY29/YKOL5078) cells. Numbers indicate cells that developed from spores of one complete tetrad. Genotypes were determined by replica plating on media containing either G418 or zeocin after 2 days of growth at 30°C. (B, C and D) Cells of the indicated genotypes (Wild type: SKY384, Δgtr1: HUY33, Δpib2: HUY34) were grown in YMM medium supplemented with ammonium sulfate, and then shifted to nitrogen-free YMM. Thirty minutes after the shift, glutamine (B and C) or leucine (D) were added at 3 mM. Phosphorylation of Sch9 (B and D) or Atg13 (C) was monitored at each time point by immunoblotting with the indicated antibodies (left panels). For the analysis of Sch9 phosphorylation, lysates were treated with NTCB and subjected to western blotting using an anti-HA antibody. Relative quantification of Sch9 or Atg13 phosphorylation is shown as the mean ± SE (n = 3 in B and D, n = 2 in C) (right panels). (E) Growth curve of Cells of the indicated genotypes (Wild type: SKY384, Δgtr1: HUY33, Δpib2: HUY34) in YMM medium supplemented with 3 mM leucine. Statistical data are shown as Mean ± SE of two independent experiments. (TIF) [file pgen.1007334.s001.tif]

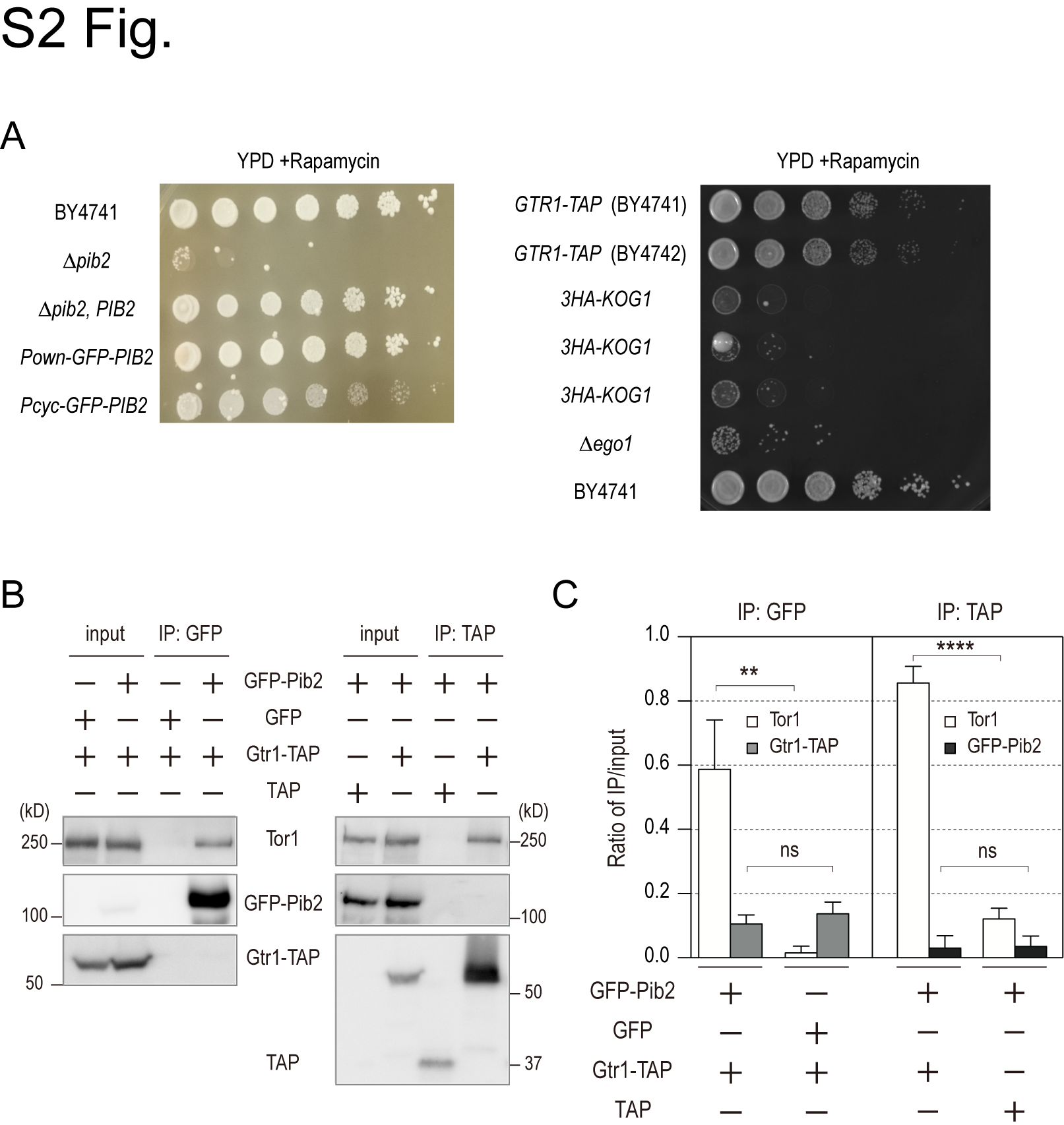

Supplement: S2 Fig — (A) Cells of the indicated genotypes were serially diluted 10-fold and spotted on YPD plates with 0.2 μg/ml rapamycin, and then grown at 30°C for 3 days. (B) Cells expressing the indicated tagged proteins were grown in SCD and analyzed as described in Fig 2A (GTR1-TAP GFP-PIB2: HUY57 with pRS316-GTR1-TAP, GTR1-TAP GFP: HUY77 with pRS316-GTR1-TAP, TAP GFP-PIB2: HUY58 with pRS316-GTR1). (C) Quantification of the ratio of IP/input in (B). Mean ± SD (n = 3). **p < 0.01, ****p < 0.0001, Student’s t-test. (TIF) [file pgen.1007334.s002.tif]

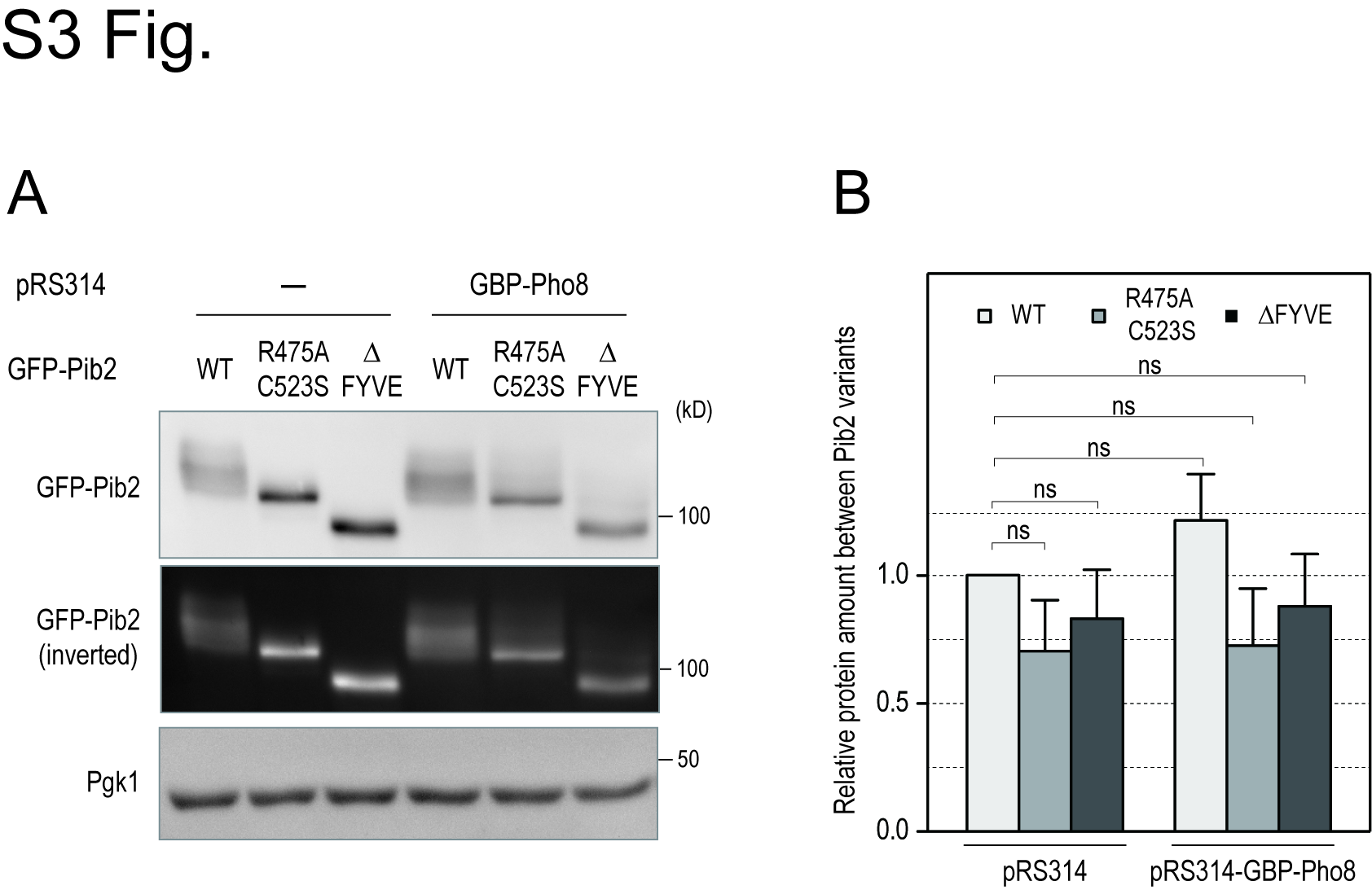

Supplement: S3 Fig — (A) Cells were cultured as described in Fig 3B. Cell extracts were prepared, then analyzed by immunoblotting with anti-GFP and anti-Pgk1 antibodies. (B) Quantification of the ratio of GFP-Pib2/Pgk1 in (A). Mean ± SD (n = 3). Significance was calculated using the student’s t-test. (TIF) [file pgen.1007334.s003.tif]

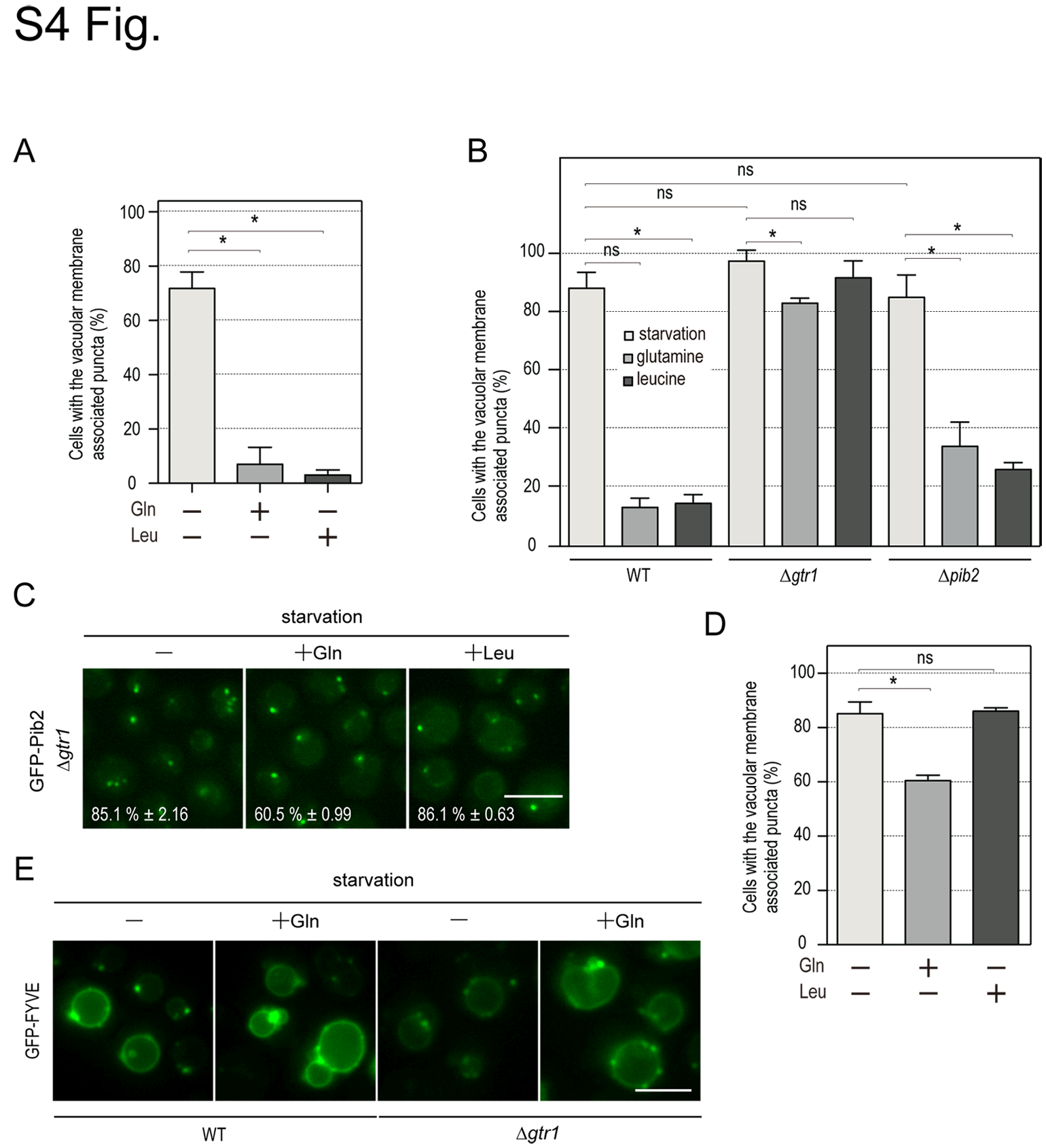

Supplement: S4 Fig — (A) Quantification of cells with vacuolar membrane associated puncta from 100–200 cells in Fig 4A. Mean ± SE (n = 4). *p < 0.05, Mann-Whitney U-test. (B) Quantification of cells with the vacuolar membrane associated puncta from 100–200 cells in Fig 4C. Mean ± SE (n = 4). *p < 0.05, Mann-Whitney U-test. (C) Cells expressing GFP-Pib2 in Δgtr1 (HUY59) were cultured and analyzed by fluorescence microscopy as in Fig 3A. Statistical data are shown as mean ± SD from 100–200 cells of four independent experiments. (D) Quantification of cells with the vacuolar membrane associated puncta from 100–200 cells in (C). Mean ± SE (n = 4). *p < 0.05, Mann-Whitney U-test. (E) Wild type (BY4741) or Δgtr1 (YKOL6522) cells harboring the GFP-FYVE plasmid (pRS425-GFP-FYVE) were cultured and analyzed by fluorescence microscopy, as in Fig 3A. (TIF) [file pgen.1007334.s004.tif]

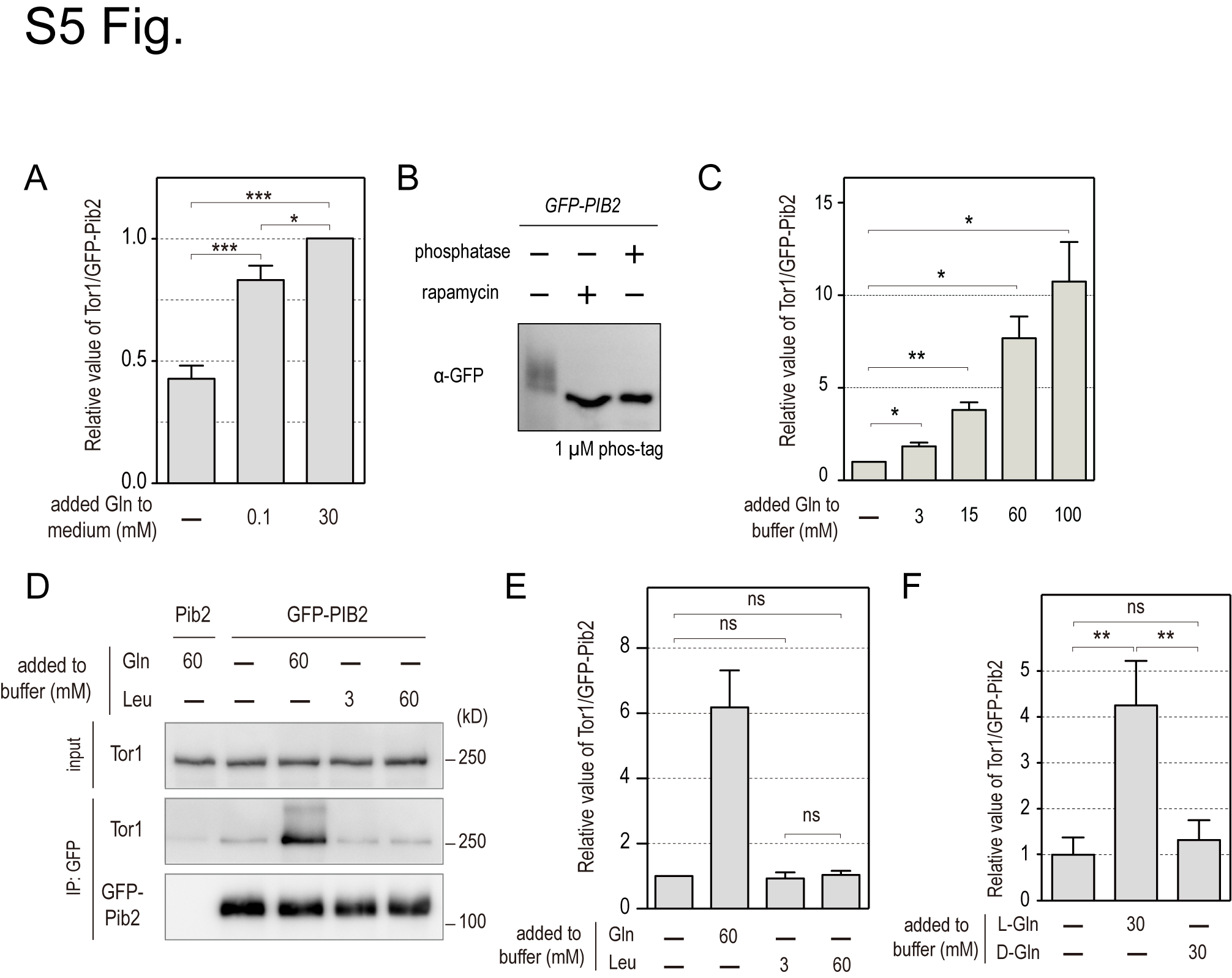

Supplement: S5 Fig — (A) Quantification of the ratio of Tor1/GFP-Pib2 in Fig 5A. Mean ± SD (n = 3). *p < 0.05, ***p < 0.001, Student’s t-test. (B) Cells grown with or without rapamycin were collected after 3 h. Immunoprecipitated GFP-Pib2 were incubated as indicated and analyzed by immunoblotting with anti-GFP antibody. (C) Quantification of the ratio of Tor1/GFP-Pib2 in Fig 4B. Mean ± SE (n = 4). *p < 0.05, Student’s t-test. (D) Cells expressing GFP-Pib2 (HUY45) were grown in YPD. Different concentrations of either l-glutamine or l-leucine were added to all buffers used in the experiment, and cell lysates were prepared and analyzed as in Fig 5A. (E) Quantification of the ratio of Tor1/GFP-Pib2 in (D). Mean ± SD (n = 3). Student’s t-test. (F) Quantification of the ratio of Tor1/GFP-Pib2 in Fig 5E. Mean ± SD (n = 3). **p < 0.01, Student’s t-test. (TIF) [file pgen.1007334.s005.tif]

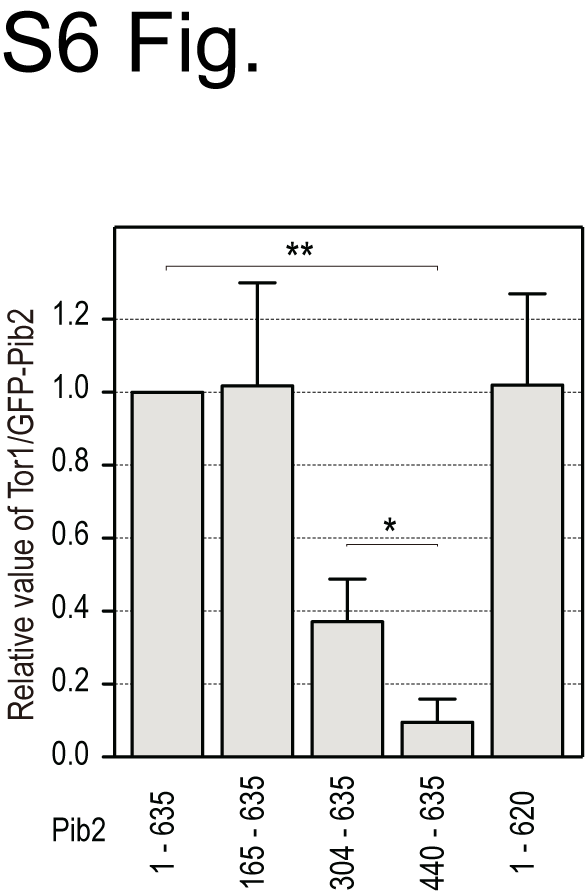

Supplement: S6 Fig — Quantification of the ratio of Tor1/GFP-Pib2 in Fig 6B. Mean ± SE (n = 3). *p < 0.05, **p < 0.01, Student’s t-test. (TIF) [file pgen.1007334.s006.tif]

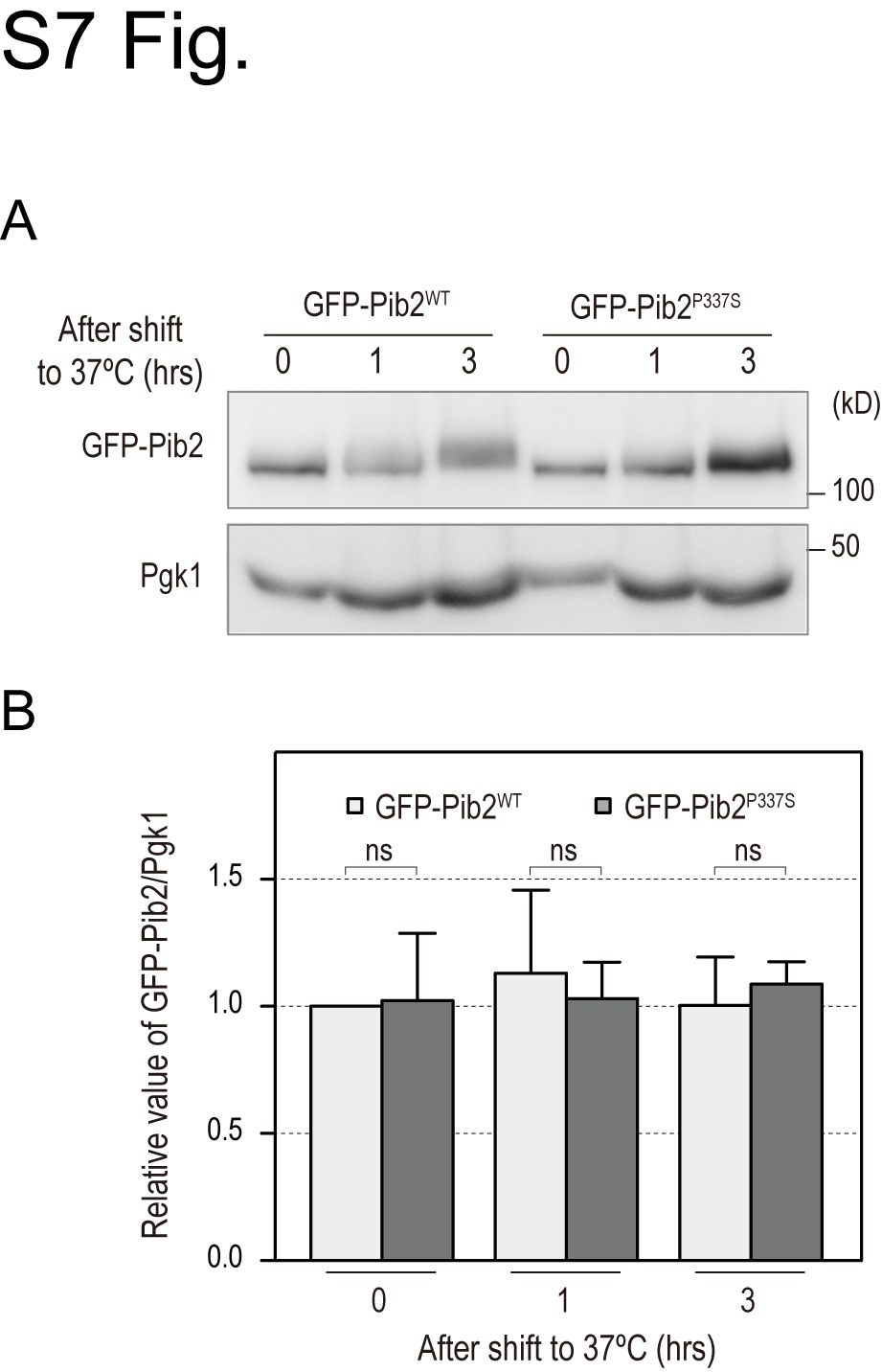

Supplement: S7 Fig — (A) Δpib2 cells expressing GFP-Pib2WT (YAY2731) and GFP-Pib2P337S (YAY2732) were grown at 30°C and then harvested. The cells were resuspended in fresh pre-warmed medium and incubated at 37°C for 1 or 3 h. Lysates were subjected to western blotting using anti-GFP and anti-Pgk1 antibodies. (B) Quantification of the ratio of GFP-Pib2/Pgk1 in (A). Mean ± SD (n = 3). Student’s t-test. (TIF) [file pgen.1007334.s007.tif]

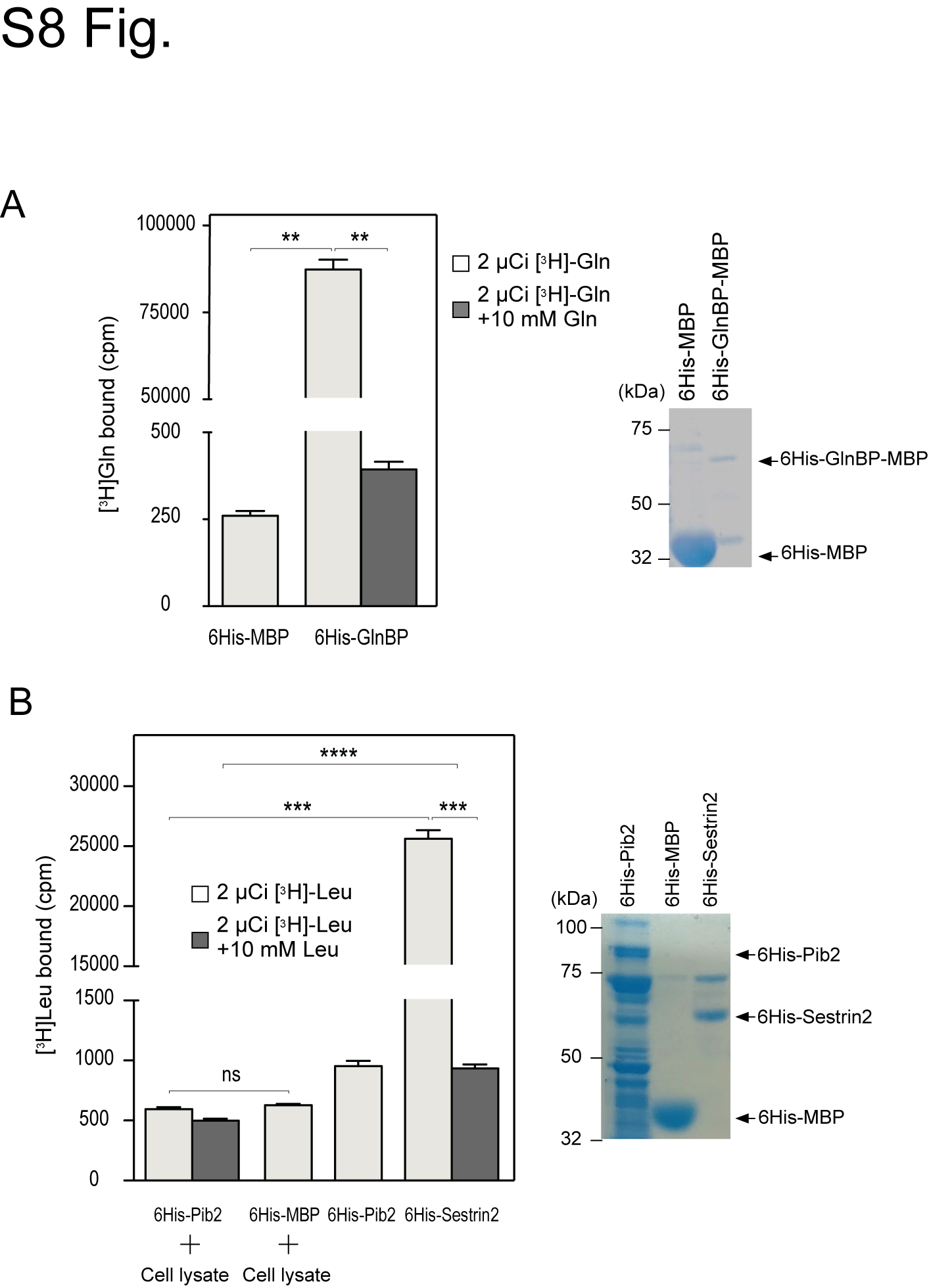

Supplement: S8 Fig — (A) Recombinant 6His-GlnBP or 6His-MBP protein on Ni-NTA agarose was incubated with [3H]l-glutamine for 30 min at 4°C. After washing, the [3H]l-glutamine-binding assay was performed as described in Materials and Methods. Unlabeled glutamine was added where indicated. Statistical data are shown as mean ± SE of three independent experiments. **p < 0.01, Student’s t-test. The purified proteins were separated on SDS-PAGE and visualized by Coomassie staining (right panel). (B) Recombinant 6His-Pib2, 6His-MBP or 6His-Sestrin2 protein on Ni-NTA agarose was incubated without or with cell lysates of Δpib2 cell (YKOL4391) for 60 min at 4°C. After washing, the [3H]l-leucine-binding assay was performed as described in Materials and Methods. Unlabeled leucine was added where indicated. Statistical data are shown as Mean ± SE of three independent experiments. ****p < 0.0001, ***p < 0.001, Student’s t-test. The purified proteins were separated on SDS-PAGE and visualized by Coomassie staining (right panel). (TIF) [file pgen.1007334.s008.tif]
