# Supplementary material for: Gtr/Ego-independent TORC1 activation is achieved through a glutamine-sensitive interaction with Pib2 on the vacuolar membrane
Source: PLoS Genet. 2018 Apr 26;14(4):e1007334. doi: 10.1371/journal.pgen.1007334 (PMC5919408; doi:10.1371/journal.pgen.1007334)
Supplement: S1 Table — (PDF) [file pgen.1007334.s010.pdf]

Table 1. List of strains used in this study

Name	Genotype	Reference
S. cerevisiae		
BY4741	*MAT*a *his3Δ1 leu2Δ0 met15Δ0 ura3Δ0*	(Brachmann et al., 1998)
BY4742	*MAT*alpha *his3Δ1 leu2Δ0 lys2Δ0 ura3Δ0*	(Brachmann et al., 1998)
FY3	*MAT*a *ura3-52*	(Winston et al.,1995)
YKOL4391	BY4741 Δ*pib2::kanMX6*	(EUROSCURF)
HUY5	BY4742 Δ*gtr1::zeoNT3*	This study
HUY29	BY4742 Δ*pib2::zeoNT3*	This study
YKOL5078	BY4741 Δ*ego1::kanMX6*	(EUROSCURF)
YKOL6522	BY4741 Δ*gtr1::kanMX6*	(EUROSCURF)
YSBN9	FY3 *ho::loxP-TEF1_Q-ble-TEF1t-loxP; ura3-52*	(Canelas et al., 2010)
SKY384	YSBN9 *SCH9-6HA::hphNT1*	This study
HUY33	SKY384 Δ*gtr1::kanMX6*	This study
HUY34	SKY384 Δ*pib2::kanMX6*	This study
HUY48	BY4741 *TETO7pr-UBI-LEU-PIB2::natNT2*	This study
HUY50	HUY48 Δ*gtr1::zeoNT3*	This study
SKY116	BY4741 *SCH9-6HA::hphNT1*	(Kira et al.,2016)
SKY118	SKY116 Δ*gtr1::kanMX6*	(Kira et al.,2016)
HUY51	HUY48 *SCH9-6HA::hphNT1*	This study
HUY53	HUY50 *SCH9-6HA::hphNT1*	This study
HUY45	BY4741 *zeoNT3::GFP-Pib2*	This study
HUY57	HUY45 *GTR1-TAP::kanMX6*	This study
SKY210	BY4741 *GTR1-TAP::kanMX6*	This study
HUY77	SKY210 *GPDpr-GFP::URA3*	This study
HUY58	HUY45 *ACTpr-TAP::kanMX6*	This study
YAY2569	BY4741 Δ*pib2::zeoNT3 GFP-PIB2*^{full}*::kanMX6*	This study
YAY2575	BY4741 Δ*pib2::zeoNT3 GFP-PIB2*$^{50\text{-}635}$*::kanMX6*	This study
YAY2570	BY4741 Δ*pib2::zeoNT3 GFP-PIB2*$^{102\text{-}635}$*::kanMX6*	This study
YAY2571	BY4741 Δ*pib2::zeoNT3 GFP-PIB2*$^{165\text{-}635}$*::kanMX6*	This study
YAY2572	BY4741 Δ*pib2::zeoNT3 GFP-PIB2*$^{304\text{-}635}$*::kanMX6*	This study

YAY2573	BY4741 Δ*pib2::zeoNT3 GFP-PIB2*$^{440-635}$*::kanMX6*	This study
YAY2574	BY4741 Δ*pib2::zeoNT3 GFP-PIB2*$^{1-620}$*::kanMX6*	This study
SKY222	BY4741 *LEU2::GFP-TOR1*	(Kira et al.,2014)
HUY39	HUY45 *VPH1-mCherry::natNT2*	This study
HUY46	HUY45 Δ*vps34::natNT2*	This study
HUY65	HUY45 Δ*atg14::hphNT1*	This study
HUY64	HUY45 Δ*vps38::hphNT1*	This study
SKY597	HUY45 Δ*atg38::natNT2*	This study
SKY598	HUY45 Δ*atg6::natNT2*	This study
HUY41	HUY45 *EGO3-mCHERRY::natNT2*	This study
SKY596	SKY222 *EGO3-mCHERRY::natNT2*	This study
SKY278	SKY222 Δ*gtr1::hphNT1*	(Kira et al.,2014)
HUY59	HUY45 Δ*gtr1::natNT2*	This study
HUY6	SKY222 Δ*pib2::zeoNT3*	This study
HUY70	BY4741 *TETO7pr-UBI-LEU-PIB2::natNT2* Δ*gtr1::zeoNT3 LEU2::GFP-TOR1*	This study
YAY2579	BY4742 Δ*pib2::zeoNT3*	This study
YAY2581	YAY2579 *kanMX4-PIB2pr-4xProA*	This study
YAY2583	YAY2579 *kanMX4-PIB2pr-4xProA-PIB2*	This study
YAY2531	BY4741 Δ*gtr1::natNT2* Δ*ego1::zeoNT3*	This study
YAY2543	YAY2531 *pib2-2-kanMX4*	This study
YAY2592	YAY2531 *SCH9-6HA::hphNT1*	This study
YAY2607	YAY2592 *pib2-2-kanMX4*	This study
SKY478	BY4741 Δ*trp1::hphNT1*	This study
HUY74	SKY478 Δ*pib2::zeoNT3 GFP-PIB2*^{full}*::kanMX6*	This study
HUY75	SKY478 Δ*pib2::zeoNT3 GFP-PIB2*$^{\Delta 440-514}$*::kanMX6*	This study
HUY76	SKY478 Δ*pib2::zeoNT3 GFP-PIB2*^{R475A,C523S}*::kanMX6*	This study
HUY71	YAY2583 *LEU2::GFP-TOR1*	This study
YAY1963	BY4741 *pACT1-TAP-NAT-leu2d0-int*	This study
YAY2724	BY4742 Δ*pib2::natNT2*	This study
YAY2731	YAY2724 *GFP-PIB2*^{full}*::kanMX4*	This study
YAY2732	YAY2724 *GFP-pib2*^{P337S}*::kanMX4*	This study

E. coli		
Rosetta2 (DE3)	*F^- ompT hsdS$_B$(r_B^- m_B^-) gal dcm (DE3) pRARE2 (Cam^R)*	(Merck)
pYA1309	Rosetta2(DE3) pET28a-*PIB2*	This study
pYA1321	Rosetta2(DE3) pMAL-c2X-6His	This study
pYA1345	Rosetta2(DE3) pET28c-hSestrin2	This study
pYA1325	Rosetta2(DE3) pMAL-c2X-GlnBP-6His	This study
